# Genetic Basis of Motor Neuron Diseases: Insights, Clinical Management, and Future Directions

**DOI:** 10.3390/ijms26104904

**Published:** 2025-05-20

**Authors:** Apostolos Antonakoudis, Stella Aikaterini Kyriakoudi, Despoina Chatzi, Iasonas Dermitzakis, Sofia Gargani, Soultana Meditskou, Maria Eleni Manthou, Paschalis Theotokis

**Affiliations:** Department of Histology-Embryology, School of Medicine, Aristotle University of Thessaloniki, 54124 Thessaloniki, Greece; antonaaa@auth.gr (A.A.); kstellaai@auth.gr (S.A.K.); chatzidc@auth.gr (D.C.); iasonasd@auth.gr (I.D.); sgargani@bio.auth.gr (S.G.); sefthym@auth.gr (S.M.); mmanthou@auth.gr (M.E.M.)

**Keywords:** motor neuron diseases, genetics, neurodegeneration, precision medicine, gene therapy

## Abstract

Motor neuron diseases (MNDs) are a heterogeneous group of neurodegenerative disorders characterized by the progressive loss of motor neurons, resulting in debilitating physical decline. Advances in genetics have revolutionized the understanding of MNDs, elucidating critical genes such as *SOD1*, *TARDBP*, *FUS*, and *C9orf72*, which are implicated in their pathogenesis. Despite these breakthroughs, significant gaps persist in understanding the interplay between genetic and environmental factors, the role of rare variants, and epigenetic contributions. This review synthesizes current knowledge on the genetic landscape of MNDs, highlights challenges in linking genotype to phenotype, and discusses the promise of precision medicine approaches. Emphasis is placed on emerging strategies, such as gene therapy and targeted molecular interventions, offering hope for personalized treatments. Addressing these challenges is imperative to harness the full potential of genomics for improving outcomes in MNDs.

## 1. Introduction

Motor neuron diseases (MNDs) represent a diverse group of progressive neurodegenerative disorders characterized by the degeneration of motor neurons in the brain and spinal cord. These conditions result in a wide range of symptoms, including muscle weakness, spasticity, and eventual paralysis, severely impairing mobility and daily functioning [1]. Patients experience symptoms that affect both upper and lower motor neurons, leading to twitching, cramps, muscle stiffness, and spasms, which further diminish coordination, walking ability, and fine motor skills. As the disease advances, speech and swallowing difficulties may emerge, further complicating patient care and quality of life [2].

The MND spectrum includes several clinically distinct disorders [3]. The most common are amyotrophic lateral sclerosis (ALS), primary lateral sclerosis (PLS), and spinal muscular atrophy (SMA), each with unique pathophysiology, symptom profiles, and disease progression rates. Additionally, less common but significant forms include infantile-onset ascending hereditary spastic paralysis (IAHSP), hereditary spastic paraplegia (HSP), progressive muscular atrophy (PMA), spinal and bulbar muscular atrophy (SBMA)—also known as Kennedy’s disease—and lethal congenital contracture syndrome (LCCS). These conditions collectively constitute the full spectrum of MNDs. Central to the pathology of MNDs are genetic mutations in several key genes, including *C9orf72*, *SOD1*, *TARDBP*, and *FUS*. These mutations contribute to motor neuron degeneration through mechanisms such as oxidative stress, impaired autophagy, and abnormal RNA metabolism. Specifically, oxidative stress leads to cellular damage, while defects in autophagy result in the accumulation of damaged proteins and organelles, disrupting neuronal homeostasis. Abnormal RNA metabolism further compromises essential cellular functions, exacerbating motor neuron degeneration [1,4,5].

This review aims to provide a comprehensive overview of the genetic foundations and molecular mechanisms underlying MNDs. Furthermore, it explores gaps in current clinical management and offers insights into potential future therapeutic strategies. By identifying the most critical pathogenetic mechanisms, this review seeks to highlight approaches for innovative therapies and precision medicine that could improve patient outcomes.

## 2. Diseases and Associated Genes

### 2.1. ALS

ALS is a progressive neurodegenerative disease that primarily affects upper and lower motor neurons in the motor cortex, brainstem, and spinal cord [6,7]. This leads to muscle weakness, atrophy, and eventual paralysis. Muscle twitching (fasciculations) and cramps are also common [8]. As the disease advances, lower motor neuron degeneration leads to muscle atrophy and weakness, while upper motor neuron damage results in spasticity and hyperreflexia [9]. Bulbar involvement occurs in about 25% of patients at onset, characterized by dysarthria (slurred speech) and dysphagia (difficulty swallowing) [10]. Respiratory muscles eventually become affected, leading to dyspnoea (difficulty breathing) and respiratory failure. Most patients with ALS die from respiratory complications within 3–5 years of symptom onset [11]. The exact cause of ALS remains unknown, but it is thought to involve both genetic and environmental factors. Several genes, including *C9orf72*, *SOD1*, *TARDBP*, and *FUS*, have been directly implicated in ALS pathogenesis. Mutations in these genes contribute to familial ALS (5–10% of cases) and sporadic ALS (90–95%) cases [12,13,14].

Apart from the genetic background, environmental risk factors are also proposed to play a part in its development. These include exposure to lead and pesticides, smoking, military service, and trauma. However, definitive causal relationships are lacking due to variability in findings. Genes associated with ALS include *SOD1*, *FUS*, *TARDBP,* and *C9orf72,* each contributing to ALS pathology through mechanisms like RNA metabolism disruption, oxidative stress, and impaired cellular trafficking, ultimately leading to motor neuron degeneration. The connection between oxidative stress and ALS is crucial, as oxidative stress is a significant contributor to the decline in cellular function in this neurodegenerative disease [15,16]. Oxidative stress occurs in cases of disparities between the production and buildup of oxygen reactive species (ROS) in cells and tissues, and the biological system’s capacity to remove these reactive substances [17]. Increased ROS concentration indicates harmful effects on cellular structures like proteins and lipids and can impair their functions and the antioxidant protection of the body [18]. In ALS, oxidative stress can lead to damage in motor neurons, accelerating their degeneration and contributing to the disease’s progression, particularly in adult-onset forms linked to aging [4,15]. The several genes, whose mutations are associated with the pathogenesis of this disease and are related to subsequent disruptions in RNA metabolism, oxidative stress, and cellular trafficking, will be analyzed.

#### 2.1.1. *SOD*

*SOD* genes are critical for protecting cells from oxidative stress. There are three known isoforms—*SOD1*, which requires copper and zinc ions to function in the cytoplasm; *SOD2*, which is manganese-dependent and located in the mitochondria; and *SOD3*, which also uses copper and zinc but localizes in the extracellular matrix. These metal ions are essential cofactors, enabling each isoform to neutralize reactive oxygen species in different cellular environments [19]. Among those three, *SOD1* plays a particularly significant role in ALS. It encodes the enzyme superoxide dismutase 1 (Cu/ZnSOD), which is crucial for converting toxic superoxide radicals into hydrogen peroxide and oxygen, helping to prevent cellular damage caused by ROS. *SOD1* functions as a homodimer, stabilized by disulfide bridges, a structure essential for its enzymatic activity. Mutations in the *SOD1* gene are directly implicated in ALS pathogenesis, leading to dysfunction of the protein, increased oxidative stress, and subsequent motor neuron degeneration [5,20]. The second isoform, *SOD2*, is located in the mitochondria, where it protects mitochondrial integrity by converting superoxide radicals into hydrogen peroxide [21]. Lastly, *SOD3* operates in extracellular spaces, where it similarly breaks down superoxide radicals, safeguarding against oxidative stress outside the cell [22].

Directly linked with *SOD1* is the Nrf2-ARE pathway, which serves as an inherent defense mechanism against oxidative stress [23]. SOD1 is intertwined with the Nrf2/ARE pathway due to its role as an antioxidant enzyme, and its expression is regulated by this pathway [24]. Nrf2 acts as a transcription factor that activates numerous genes, such as NQO1 (NAD(P)H quinone dehydrogenase 1), HO-1 (Heme oxygenase 1), GPX (Glutathione peroxidase), and CAT (Catalase), which are responsible for protecting cells and aiding in detoxification processes [25,26,27]. Upon activation, the Nrf2 pathway upregulates the transcription of *SOD1*, increasing *SOD1* protein levels and amplifying the cell’s antioxidant capacity, thereby enhancing its ability to combat oxidative stress [28]. Furthermore, Nrf2 is increasingly recognized for its role in regulating mitochondrial function, particularly through mitophagy, a process critical for removing damaged mitochondria and preventing mitochondrial dysfunction and oxidative stress accumulation. Nrf2 also intersects with inflammatory signaling pathways, especially through the inhibition of the NF-κB pathway, highlighting its dual role in reducing oxidative stress and inflammation, both of which are implicated in ALS progression [29,30]. Dysfunction in either the *SOD1* gene or the Nrf2/ARE signaling pathway may contribute to ALS by weakening cellular defenses against oxidative stress and inflammation, accelerating motor neuron degeneration [31].

Several mechanisms have been suggested to explain the contribution of oxidative stress in the development of MNDs, with two being the most prominent. Specifically, the first mechanism involves mutations in *SOD1* that induce structural changes leading to cytotoxic enzymatic activity and increased oxidative stress [32]. Mutations in the *SOD1* gene, such as G93A and A4V, alter the enzyme’s structure in ways that directly impair its ability to neutralize superoxide radicals, resulting in a toxic buildup of ROS. These mutations disrupt the enzyme’s metal-binding sites, which are crucial for its normal catalytic activity. As a result, mutant *SOD1* is less effective at neutralizing superoxide radicals, and this loss of function leads to an accumulation of superoxide radicals, significantly elevating oxidative stress within cells [33,34]. The resulting oxidative stress damages vital cellular components like proteins, lipids, and DNA. Neurons are particularly susceptible to oxidative stress due to their high energy demands and limited repair capacity. This vulnerability makes oxidative stress a central factor in MNDs, particularly in ALS, where it contributes to neuronal degeneration and the progressive loss of motor function [35]. The second mechanism involves *SOD1* mutations that increase the likelihood of protein misfolding and aggregation, which disrupt cellular processes and contribute to neurodegeneration [36]. Additionally, these structural changes lead to the formation of cytoplasmic inclusions. The aggregation of misfolded *SOD1* variants contributes significantly to cellular toxicity. These aggregates impair mitochondrial function, which not only reduces ATP production but also increases the generation of ROS. Furthermore, *SOD1* aggregates disrupt axonal transport, crucial for maintaining neuronal health and function, thereby compounding the oxidative damage within motor neurons and accelerating their degeneration [37,38,39].

Recent studies have expanded the understanding of ALS pathogenesis by highlighting the role of non-neuronal cells, particularly glial cells and muscle cells, in the disease. Mutant *SOD1* not only impacts motor neurons but also creates a toxic environment in surrounding glial cells, which become hyperreactive due to the activation of inflammatory pathways [40]. This activation triggers microglia and astrocytes to release proinflammatory cytokines, such as TNF-α, IL-1β, and IL-6, leading to increased oxidative stress and excitotoxicity, contributing to neurotoxicity and accelerating motor neuron degeneration. This triggers an inflammatory response, contributing to neurotoxicity and accelerating motor neuron degeneration [41,42]. Neuroinflammation is critical for understanding how *SOD1* mutations contribute to the progression of ALS. Glial cells, normally responsible for supporting neurons and maintaining a healthy extracellular environment, become reactive in the presence of mutant *SOD1*, amplifying the neurotoxic environment [43]. Astrocytes, which typically regulate extracellular glutamate and provide metabolic support to neurons, become reactive when exposed to mutant *SOD1*. This reactivity leads to the release of proinflammatory cytokines and a failure to clear excess glutamate, resulting in excitotoxicity. The resulting neuroinflammatory state exacerbates oxidative stress and accelerates neuronal death, illustrating how glial cells contribute to *SOD1*-driven disease mechanisms [44].

Similarly, microglia are heavily implicated in this process. Mutant *SOD1* triggers microglia to shift from a neuroprotective role to a proinflammatory, neurotoxic state. Activated microglia release additional ROS and proinflammatory molecules, further damaging motor neurons. The interaction between reactive microglia and mutant *SOD1*-expressing neurons creates a vicious cycle of inflammation and oxidative stress, underscoring the importance of targeting both neuronal and glial dysfunction in therapeutic strategies for ALS [45]. Additionally, skeletal muscle cells show early signs of dysfunction due to calcium dysregulation, particularly in the context of mutant *SOD1*. This dysregulation arises from impaired calcium handling mechanisms, including alterations in calcium release from the sarcoplasmic reticulum and disruptions in calcium channels. The altered calcium homeostasis impacts mitochondrial function, further increasing ROS production and impairing the cross-talk between motor neurons and muscle cells. Disruptions in calcium signaling impair mitochondrial calcium regulation, leading to energy deficits and contributing to muscle weakness in ALS [46].

#### 2.1.2. *TARDBP*

Defects in RNA metabolism and processing are also implicated in ALS development [47]. ALS has been increasingly linked to mutations in genes encoding RNA-binding proteins. *TARDBP* is one of these genes. Specifically, *TARDBP* encodes the TDP-43 protein (TAR DNA-binding protein 43), which plays a crucial role in various essential cellular processes, including transcription, splicing, RNA transport, and stability. In its normal state, *TARDBP* forms RNA transport granules that guide mRNA molecules to the dendrites and axons for local translation, enabling neurons to rapidly respond to synaptic activity [48,49]. This localization supports the synthesis of proteins required for synaptic remodeling, which is vital for learning, memory, and overall cognitive function by binding to RNA molecules with UG-rich sequences via its RNA recognition motifs (RRMs), TDP-43 regulates the lifespan and transport of specific mRNAs, which is particularly important in neurons where localized translation supports synaptic function and plasticity. Through these interactions, TDP-43 ensures proper mRNA localization and protein synthesis in response to cellular signals [50].

Additionally, *TARDBP* plays a crucial role in regulating protein synthesis within dendrites. In neurons, localized protein synthesis is essential for synaptic function and plasticity. By controlling the transport of specific mRNAs to dendrites and facilitating their local translation, *TARDBP* ensures that proteins required for synaptic signaling and remodeling are synthesized close to their site of action. This localized control of protein synthesis allows neurons to rapidly adapt to changes in synaptic activity, maintaining their functional integrity [51]. *TARDBP* helps manage this process by interacting with RNA-binding proteins and ribonucleoprotein complexes that regulate translation. TDP-43 is vital for maintaining normal neuronal function by regulating RNA metabolism and protein homeostasis by preventing toxic aggregate formation [52].

During cellular stress, it is also involved in the formation and regulation of stress granules, which are structures that pause translation and stabilize RNA, allowing the cell to manage stress until conditions normalize. Stress granules act as temporary storage sites for stalled translation initiation complexes, helping the cell conserve resources and manage stress. When participating in stress granule formation, *TARDBP* helps cells adapt to stress by pausing translation and protecting RNA from degradation [53,54]. Once the stress reduces, stress granules disassemble, allowing normal cellular function to resume. Mutations in *TARDBP* have been identified as significant contributors to familial ALS. Among these mutations, three notable heterozygous missense mutations have been characterized, which will be mentioned in the following paragraphs. Missense mutations are the most common mutations in *TARDBP* and are characterized by a single amino acid change that can significantly alter the protein’s ability to bind RNA. This disruption can lead to improper RNA processing and the aggregation of TDP-43 in the cytoplasm, contributing to neurodegenerative processes.

Notably, mutations in the C-terminal glycine-rich domain of TDP-43 are particularly impactful, as this region is critical for protein–protein interactions, further exacerbating the pathogenic effects associated with these mutations [55,56]. Firstly, p.M337V involves a substitution of methionine for valine at codon 337, which has been reported in familial ALS cases. The mutation affects a highly conserved region of TDP-43, which is crucial for its interactions with other proteins involved in RNA processing. Biochemical studies suggest that the presence of this mutation may lead to altered protein stability and function, contributing to neurodegenerative processes [57,58]. Secondly, p.N345K, which represents a mutation where asparagine is replaced by lysine at codon 345, introduces a positive charge to the protein structure, which may disrupt TDP-43’s normal biochemical interactions. The functional consequences of this mutation are still under investigation, but initial findings indicate that it may influence the aggregation propensity of TDP-43, further exacerbating its neurotoxic effects [55,59].

p.I383V is another heterozygous missense mutation that substitutes isoleucine for valine at codon 383. Similar to the other mutations, this alteration occurs within the C-terminal domain of TDP-43, which is essential for its protein–protein interactions. The effects of this mutation on TDP-43 functionality are not yet fully understood, but its presence in familial ALS patients suggests it may also lead to a toxic gain of function and contribute to motor neuron degeneration [56,60]. Biochemical analyses of lymphoblastoid cell lines derived from ALS patients presenting with these mutations have revealed a substantial accumulation of caspase-cleaved TDP-43 fragments, specifically at approximately 35 kDa and 25 kDa. This accumulation indicates a toxic gain of function that may lead to cellular apoptosis and subsequent motor neuron degeneration [60]. Importantly, these mutations were not found in a large cohort of control individuals, reinforcing their significance as rare but critical genetic factors in familial ALS. The frequency of *TARDBP* mutations in familial ALS patients is low, highlighting the importance of genetic screening in this patient population [61]. Continued research into the functional implications of these mutations and their effects on TDP-43 dynamics is essential for developing targeted therapeutic strategies aimed at mitigating the consequences of TDP-43 pathology in ALS.

The mutations mentioned above are linked to TDP-43 aggregation and mislocalization. In ALS, TARDBP mislocalizes from the nucleus to the cytoplasm, forming pathological aggregates that disrupt RNA processing and transcription regulation. This mislocalization results from impairments in nuclear transport and dysregulation of nuclear export signals (NESs), exacerbated by oxidative stress [62]. Cytoplasmic TARDBP accumulation is driven by dysfunction in protein degradation pathways, including the ubiquitin–proteasome system (UPS) and autophagy. Its presence in stress granules suggests a broader cellular stress response, which, if unresolved, becomes pathogenic [63]. Mislocalized TDP-43 both loses nuclear functions and gains toxic cytoplasmic functions, leading to aberrant protein interactions, impaired homeostasis, and activation of apoptotic pathways [64]. Additionally, TARDBP mislocalization disrupts mRNA transport and local protein synthesis necessary for synaptic plasticity, weakening neuronal communication and contributing to degeneration. Persistent stress granules further dysregulate RNA metabolism, increasing cellular stress and promoting aggregation, ultimately exacerbating neurodegeneration in MNDs [65].

TDP-43 has been shown to undergo liquid–liquid phase separation (LLPS), a process by which proteins form dynamic, membraneless condensates essential for normal cellular function [66]. Through LLPS, TDP-43 contributes to the assembly of structures such as stress granules, paraspeckles, and nucleoli. This phase separation is mediated by its prion-like domain (PrLD) and RNA-binding domains, which provide the multivalent interactions necessary for condensate formation. However, dysregulation of TDP-43 LLPS, often influenced by disease-linked mutations, can drive aberrant phase transitions from liquid-like states to solid, pathogenic aggregates. Such aggregation disrupts cellular homeostasis and is considered a critical mechanism underlying motor neuron degeneration in ALS [67]. Table 1 summarizes key genes implicated in ALS pathogenesis, highlighting their encoded proteins, chromosomal loci, and primary pathogenic mechanisms based on current molecular insights.

#### 2.1.3. *FUS*

FUS, which is encoded by the *FUS* gene, is a vital RNA-binding protein that plays a significant role in multiple aspects of RNA metabolism and processing [73]. Dysfunctions of this protein or mutations in *FUS* are linked to ALS [74,75]. Primarily located in the nucleus, FUS is responsible for RNA binding and processing, particularly in the 3′ untranslated regions (UTRs) of mRNA [76,77]. Through its interaction with the transcription complex, FUS plays a key role in the regulation of gene expression, ensuring the proper splicing and maturation of mRNA. It also facilitates the transport of RNA molecules to specific subcellular compartments, an essential function for maintaining cellular homeostasis [47,78].

Mutations in *FUS* can arise due to various mechanisms, including spontaneous errors in DNA replication, genetic inheritance, and environmental factors. Spontaneous mutations may occur due to errors during DNA replication or repair processes, leading to alterations in the protein’s structure and function. Hereditary mutations in *FUS* can be passed through generations, particularly affecting individuals with familial forms of neurodegenerative diseases like ALS. These mutations lead to both toxic gain-of-function and loss-of-function effects, disrupting key cellular processes and contributing to disease pathology. Additionally, environmental factors, such as exposure to oxidative stress or toxins, can induce DNA damage, resulting in mutations in *FUS*. Mutations in *FUS* can cause the protein to mislocalize to the cytoplasm. This mislocalization results in a loss-of-function effect, as the FUS protein is no longer available to perform its critical nuclear tasks [79,80]. The mislocalized FUS protein cannot effectively regulate RNA processing and transcriptional activities, leading to defects in RNA maturation and processing. The loss of these nuclear functions results in the accumulation of defective or unprocessed RNA species, which impairs normal gene expression and cellular function [81]. The effect of the mislocalized FUS extends to RNA metabolism, where the protein’s inability to participate in the splicing of pre-mRNA and the assembly of ribonucleoprotein complexes leads to further accumulation of improperly processed RNA. This disruption in RNA processing is a key factor in the pathogenesis of diseases associated with *FUS* mutations [82].

Furthermore, mutant FUS molecules are prone to forming cytoplasmic aggregates, a common sign of various neurodegenerative diseases. These aggregates consist of misfolded or aberrantly processed FUS proteins that accumulate in the cytoplasm of affected neurons [81]. This misfolding can be considered to exhibit a gain-of-function effect due to the mutant *FUS* proteins acquiring toxic properties, leading to the formation of aggregates that disrupt normal cellular function [83]. The cytoplasmic inclusions formed by these aggregates are composed of hyperphosphorylated and ubiquitinated FUS, indicating a cellular stress response to abnormal protein accumulation. The formation of these cytoplasmic aggregates leads to multiple detrimental consequences. Essential proteins and RNA molecules are separated by the aggregates, impairing their availability and functionality. Crucial cellular processes, including RNA metabolism, protein synthesis, and cellular signaling, are disrupted as a result. Additionally, the aggregates introduce a toxic gain of function, interfering with normal cellular activities while triggering oxidative stress and inflammation [84,85]. This, in turn, exacerbates cellular toxicity and contributes to disease progression [83]. Additionally, the aggregation of FUS proteins can trigger stress-related signaling pathways and inflammatory responses, further contributing to neuronal dysfunction and neurodegeneration. Regarding the two mechanisms, aggregation introduces toxic gain-of-function effects that disrupt cellular processes, while mislocalization results in a loss of function that impairs essential RNA processing activities [83]. Together, these mechanisms lead to neuronal dysfunction, cellular stress, and inflammation, which are characteristic features in MNDs [86].

*FUS* mutations are associated with early-onset ALS, often leading to a more aggressive disease course [87]. This correlation was further confirmed in a recent study, which highlighted that early-onset cases with *FUS* mutations exhibit rapid disease progression and shorter survival rates compared to mid-to-late-onset cases. Patients with *FUS* mutations typically experience rapid motor function decline, resulting in shorter survival times compared to sporadic ALS cases. The distinct clinical presentation of *FUS*-linked ALS highlights the need for genetic testing in younger patients with ALS symptoms [68,88]. Genetic testing for *FUS* mutations is crucial for diagnosing ALS, particularly in patients with a family history of these diseases. Early identification of *FUS* mutations can help in diagnosing younger patients and guide treatment strategies, as the clinical course of *FUS*-related diseases can be more aggressive. Given this strong clinical correlation, genetic testing for FUS mutations is important for early diagnosis, informing prognosis, and guiding patient management, including genetic counseling [89].

#### 2.1.4. *C9orf72*

The *C9orf72* gene encodes a protein that plays an important role in cellular homeostasis. This gene is associated with neurodegenerative disorders, particularly ALS. *C9orf72* has various roles in maintaining cellular homeostasis, through its role in autophagy, which is critical for clearing toxic proteins and preventing neurodegeneration, particularly in neurons [90,91]. Additionally it is involved in endocytosis, autophagy, and lysosomal function [92,93,94]. Mutations in *C9orf72* disrupt autophagy by reducing C9orf72 protein levels and producing toxic dipeptide repeats, which impair autophagic flux and lysosomal function, contributing to neurodegeneration in ALS and other MNDs [95]. Additionally, C9orf72 interacts with RNA-binding proteins involved in RNA splicing, transport, and stability, which are crucial for the proper processing and maturation of mRNA necessary for accurate gene expression and protein synthesis.

Disruptions in RNA processing due to *C9orf72* mutations can lead to the accumulation of toxic proteins. Furthermore, C9orf72 plays a role in the stress response by assisting in the formation of stress granules, which protect RNA and proteins under cellular stress [96,97]. Mutations in *C9orf72* can impair stress granule formation, increasing neuronal vulnerability and accelerating neurodegeneration in ALS [98]. The discovery of hexanucleotide repeat expansions (G4C2 repeats) within this gene marked a major breakthrough, revealing that these mutations are the most prevalent genetic cause of familial ALS [99]. These repeat expansions are characterized by an abnormal increase in the number of G4C2 sequences within the gene, which leads to various pathogenic mechanisms that drive the neurodegenerative processes seen in ALS [69]. In individuals affected by ALS, the number of G4C2 repeats can extend into the hundreds or thousands, while unaffected individuals typically have fewer than 30 repeats. This expanded repeat region has been linked to several pathogenic mechanisms that contribute to neurodegeneration. One major mechanism is RNA toxicity. The repeat expansions form abnormal RNA foci in neurons, which sequester RNA-binding proteins, leading to disruptions in RNA metabolism. This interference in normal cellular processes contributes to the degeneration of motor neurons in ALS [70,100,101].

Additionally, another key mechanism involves protein aggregation. The repeat expansions result in the production of dipeptide repeat (DPR) proteins via non-ATG translation, which accumulate in neurons and form toxic aggregates. These aggregates further contribute to cellular dysfunction and neurodegeneration [102]. Understanding the specific ways in which these mutations disrupt cellular functions is essential for developing targeted therapeutic strategies and improving patient outcomes in these devastating diseases.

#### 2.1.5. *ALS2*

*ALS2*, which encodes the protein Alsin, plays a crucial role in endosomal trafficking and autophagy. Alsin plays a key role in maintaining the health and function of motor neurons by facilitating the proper recycling and degradation of cellular components [103,104]. Alsin participates in Rab5-mediated endosome fusion, a process essential for the proper function of the endocytic pathway. This ensures efficient intracellular trafficking, allowing for the correct sorting and processing of proteins and lipids [105]. *ALS2* also plays a crucial role in autophagy, aiding in the clearance of damaged proteins and organelles. This function is particularly important for neuron health, as the accumulation of cellular debris can lead to neurodegenerative conditions [106]. Finally, *ALS2* is involved in vesicle transport, which is vital for maintaining axonal integrity by facilitating the movement of vesicles within neurons, thereby preserving both the structure and function of motor neurons [107,108].

Mutations in *ALS2* result in impaired Alsin function, disrupting cellular homeostasis and contributing to motor neuron degeneration. Mutations in the *ALS2* gene, i.e., nonsense, frameshift, and missense mutations, often lead to premature truncation or single amino acid substitutions in the Alsin protein. One of the primary consequences of these mutations is the impairment of endosomal trafficking [109]. Without functional Alsin, the *ALS2*-mediated endosomal trafficking pathway becomes dysfunctional, leading to the improper transport and degradation of proteins and organelles within the neuron [108,110]. This disruption has a cascading effect on other vital neuronal processes, particularly autophagy. The disruption of autophagy is a central pathological feature of *ALS2* mutations [111,112]. However, when Alsin is lost or its function is compromised, this autophagic process becomes defective. Damaged cellular components accumulate within motor neurons due to inefficient clearance, creating a toxic environment that triggers neuronal death. Moreover, defective autophagy renders neurons highly susceptible to oxidative stress [113,114]. In neurons lacking functional Alsin, the impaired clearance of these damaged structures results in elevated oxidative stress, further contributing to the degeneration of motor neurons. This oxidative damage accelerates neurodegenerative processes, worsening the progression of MNDs [115,116]. Another significant pathological consequence of *ALS2* mutations is the impairment of vesicle transport. The failure of Alsin/Rab5 GTPase interaction results in the disruption of the delivery of essential molecules along axons, leading to neuronal dysfunction.

Alsin contains several functional domains that mediate its diverse cellular activities. The N-terminal RCC1-like domain (RLD) facilitates guanine nucleotide exchange, contributing to intracellular signaling. The central DH/PH tandem domain regulates Rho GTPases and is involved in cytoskeletal organization and endosomal trafficking. The VPS9 domain promotes Rab5 activation, which is essential for early endosome function, while the multiple MORN motifs are associated with membrane binding [110,117]. Mutations affecting these domains impair vesicle trafficking, membrane dynamics, and cytoskeletal regulation, ultimately promoting motor neuron degeneration in conditions such as juvenile ALS and hereditary spastic paraplegia. Domain-specific analysis offers a useful framework for linking molecular defects in Alsin to clinical phenotypes and may guide future therapeutic strategies [118].

The most prominent condition linked to *ALS2* mutations is juvenile-onset ALS, a rare form of ALS that typically manifests before the age of 25. Unlike adult-onset ALS, juvenile-onset ALS tends to progress more slowly but still leads to the degeneration of both upper and lower motor neurons. The impaired Alsin function in juvenile-onset ALS results in the disruption of neuronal repair and maintenance, causing progressive muscle weakness, spasticity, and difficulties with movement and coordination. Over time, patients may experience significant motor impairment, including issues with speech and swallowing, although cognitive function generally remains intact, indicating that the neurodegenerative effects of Alsin dysfunction are primarily motor-focused [119,120]. Understanding these mechanisms is essential for improving patient outcomes in these devastating diseases.

#### 2.1.6. *ATAXIN-2*

*ATXN2* has also been implicated in the pathology of ALS and Spinocerebellar Ataxia Type 2 (SCA2). Mutations in *ATXN2*, particularly expansions in its polyglutamine (polyQ) domain, are associated with an increased risk of ALS. *ATXN2* interacts with RNA-binding proteins like TDP-43 and contributes to RNA metabolism and stress granule dynamics. Expanded polyQ repeats in *ATXN2* can enhance TDP-43 aggregation and disrupt cellular homeostasis, further contributing to disease mechanisms. Understanding *ATXN2’s* role offers additional insights into RNA metabolism dysfunction in MNDs [121,122,123].

### 2.2. SMA

Spinal muscular atrophy (SMA) is a genetic disorder characterized by the degeneration of lower motor neurons in the spinal cord, resulting in progressive muscle weakness and atrophy [124]. SMA is classified into four main types, each varying in severity and age of onset. Common symptoms across all types include difficulty with swallowing, breathing, and loss of mobility. Weakness is more pronounced in proximal muscles [125]. Type 1, the most severe form, manifests in infancy with profound muscle weakness, difficulty breathing, and trouble swallowing. Without treatment, these symptoms often lead to early mortality due to respiratory failure [126,127]. Type 2 affects children between 6 and 18 months of age. Patients with this form can sit independently but are unable to stand or walk unaided, with muscle weakness and respiratory issues being prominent, although life expectancy is longer than in Type 1 [128]. Type 3 develops after 18 months, usually in childhood. Individuals can often walk during early years but may lose this ability over time as weakness progresses [129]. Type 4, the mildest form, has an onset in adulthood, with slower disease progression and less severe muscle weakness [130]. These observations further support the view that the neurodegenerative effects of Alsin dysfunction are primarily motor-focused [119,120], and understanding these mechanisms is crucial for improving patient outcomes in these devastating diseases.

#### *SMN1/SMN2* 

**Figure 1:**
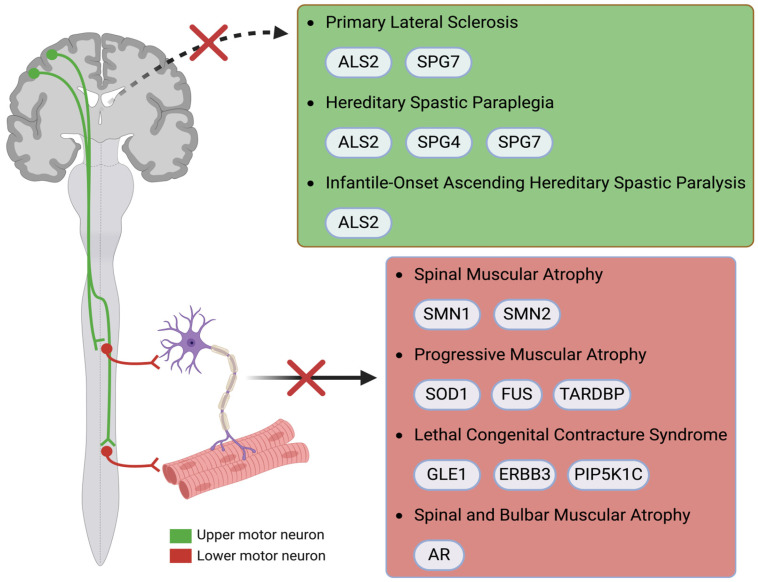
Genes implicated in various MNDs. In PLS, HSP, and IASHP, upper motor neurons are affected, while in SMA, PMA, LCCS, and SBMA, lower motor neurons are affected. ALS: amyotrophic lateral sclerosis; ALS2: amyotrophic lateral sclerosis 2; AR: androgen receptor; ERBB3: Erb-B2 receptor tyrosine kinase 3; FUS: Fused in Sarcoma; GLE1: GLE1 RNA export mediator; HSP: hereditary spastic paraplegia; IAHSP: infantile-onset ascending hereditary spastic paraplegia; LCCS: lethal congenital contracture syndrome; MND: motor neuron disease; PIP5K1C: phosphatidylinositol-4-phosphate 5-kinase type I gamma; SBMA: spinal and bulbar muscular atrophy; SMA: spinal muscular atrophy; SMN: survival motor neuron; SOD1: superoxide dismutase 1; SPG4: spastin; SPG7: paraplegin; TARDBP: TAR DNA-binding protein gene.

SMA is caused by mutations in the *SMN1* gene, which encodes the survival motor neuron (SMN) protein [131]. The clinical severity of SMA varies, primarily depending on the number of functional *SMN2* gene copies. While *SMN2* can produce some SMN protein, it does so inefficiently, leading to disease modification but not prevention. Individuals with more copies of *SMN2* tend to have milder forms of SMA [132]. *SMN1* encodes the survival motor neuron (SMN) protein, which is essential for the maintenance and function of motor neurons [133]. SMN is a critical component of the spliceosome, the molecular machinery responsible for processing pre-mRNA into mature mRNA. SMN plays an essential role in assembling small nuclear ribonucleoproteins (snRNPs), which are required for accurate and efficient splicing. By ensuring the proper removal of introns and the joining of exons, SMN facilitates the generation of mature mRNA, enabling the synthesis of proteins necessary for various cellular functions. This process is fundamental for the regulation of gene expression and the maintenance of normal cellular activity (Figure 1) [134].

Additionally, the SMN protein plays a vital role in axonal transport. Axons, which can extend long distances from the cell body, require an efficient transport system to deliver critical components such as organelles, proteins, and mRNA to the distal parts of the neuron. SMN is involved in facilitating this transport, ensuring that neurons receive the necessary materials to maintain their structure, function, and communication with other cells. Proper axonal transport is crucial for the health and functionality of motor neurons, allowing them to sustain long-term activity and support motor control [135,136,137]. Finally, the SMN protein helps maintain the integrity of cellular processes by preventing the accumulation of damaged proteins and organelles. By facilitating the efficient transport and processing of cellular components, SMN ensures that unnecessary or malfunctioning proteins are properly degraded and recycled. This regulation is essential for neurons, which are highly sensitive to the buildup of damaged materials [138]. Through its role in maintaining protein balance, SMN contributes to cellular health and reduces the risk of protein aggregation, supporting the long-term functionality of motor neurons.

*SMN1* is associated with SMA due to mutations that disrupt its function. These mutations are inherited in an autosomal recessive manner, with the most common alteration being the deletion of the entire *SMN1* gene, leading to the complete loss of functional SMN protein [139]. In addition to gene deletions, point mutations can occur that disrupt the gene’s ability to produce a functional protein [140]. These genetic alterations impair the essential roles of the SMN protein, ultimately resulting in the degeneration of motor neurons and the clinical manifestations of SMA. Insufficient levels of SMN protein lead to the degeneration of motor neurons in the spinal cord and brainstem, resulting in muscle weakness and atrophy. This loss of motor neurons is often exacerbated by apoptosis [141]. In this context, several apoptotic pathways may be activated in response to low SMN protein levels. One key pathway involves the intrinsic mitochondrial mechanism, where cellular stressors trigger the release of pro-apoptotic factors, like cytochrome c, from the mitochondria. This release activates caspases, essential cysteine proteases involved in the apoptotic process [142]. Additionally, the activation of the p53 tumor suppressor protein may occur in response to DNA damage or cellular stress, further promoting apoptosis [143]. The combined effects of these pathways contribute to the progressive loss of motor neurons, impairing motor function and overall neuronal health.

Following the loss of motor neurons, insufficient SMN protein levels also impair essential cellular functions like RNA splicing and axonal transport. RNA splicing, necessary for mRNA maturation, depends on the assembly of snRNPs, which is compromised by low SMN levels, leading to improper protein synthesis. Additionally, axonal transport is disrupted, hindering the delivery of critical materials along motor neuron axons. These impairments collectively diminish the health and functionality of motor neurons, contributing to their degeneration. Furthermore, impaired SMN function disrupts cellular homeostasis, resulting in the accumulation of damaged proteins and organelles within motor neurons. This accumulation occurs when the mechanisms for degrading and recycling cellular components are compromised [134,144,145]. The number of copies of *SMN2* directly impacts disease severity. Each additional copy of *SMN2* increases the production of the SMN protein, which can partially mitigate the effects of *SMN1* deficiency. Although *SMN2* cannot fully compensate for the loss of *SMN1*, its presence is crucial for improving motor neuron health and function, thereby influencing the clinical manifestations and progression of SMA [146,147].

### 2.3. PLS

Primary lateral sclerosis (PLS) is a rare neurodegenerative disorder characterized by the degeneration of upper motor neurons in the brain and brainstem [148]. The symptoms are muscle stiffness, spasticity, and weakness. PLS often presents initially with difficulty in leg movements, followed by symptoms such as dysarthria and dysphagia [149]. Unlike ALS, which involves both upper and lower motor neurons, PLS is confined to upper motor neuron degeneration. The onset of PLS is typically slower, and although it results in significant disability, it progresses less rapidly than ALS. In contrast to ALS, PLS generally does not lead to significant muscle atrophy or respiratory failure, and most individuals with PLS have a normal life expectancy [150]. However, the progressive loss of voluntary motor control significantly impacts quality of life, leading to physical disability and limitations in daily activities. Recent studies suggest that while PLS is primarily considered sporadic, these genetic associations highlight the need for further exploration into hereditary forms of the disease, potentially aiding in earlier diagnosis and therapeutic interventions.

While PLS is not as strongly associated with genetic mutations, some cases have been linked to mutations in *ALS2*, which encodes the protein Alsin, which is vital for regulating intracellular processes and especially endosomal trafficking, autophagy, and membrane dynamics. Additionally, recent research has identified potential associations between PLS and mutations in other genes, including *SPG7*, which encodes paraplegin, a mitochondrial protein involved in maintaining mitochondrial function. The loss of paraplegin leads to mitochondrial dysfunction, which is increasingly recognized as a key factor in neurodegeneration [151]. In PLS, the loss of Alsin disrupts endosomal trafficking and autophagy in cortical motor neurons. This impairment contributes to the variety of features of PLS, including spasticity, muscle stiffness, and gradually worsening motor control [152]. Unlike ALS, PLS progresses more slowly and does not usually involve lower motor neuron degeneration, sparing muscles from atrophy and respiratory failure, though it still results in significant disability over time. The longer life expectancy in PLS compared to ALS may reflect the selective vulnerability of upper motor neurons to Alsin dysfunction, as opposed to the combined upper and lower motor neuron degeneration seen in ALS [152,153]. The clinical presentation and progression of *ALS2*-related MNDs can vary significantly depending on the specific mutation and its impact on neuronal function [154]. Some mutations result in slower neurodegeneration, as seen in juvenile-onset ALS and PLS, while others, like those in IAHSP, lead to a rapid decline due to early and severe motor neuron damage. This variability underscores the importance of understanding the molecular mechanisms behind each mutation to tailor therapeutic strategies effectively.

### 2.4. IAHSP

Infantile-onset ascending hereditary spastic paralysis (IAHSP) is a rare MND characterized by the progressive degeneration of upper motor neurons, leading to spasticity and muscle weakness, primarily in the lower limbs. In IAHSP, symptoms arise in infancy due to the early impact of *ALS2* mutations on upper motor neurons, particularly in the corticospinal tract [155]. In this disorder, the lack of functional Alsin leads to severe disruptions in intracellular transport mechanisms, resulting in rapid and progressive spasticity and weakness, primarily in the lower limbs, which gradually ascend to involve the upper limbs [156]. This condition often leads to severe motor impairments early in childhood, with profound limitations on voluntary motor control. IAHSP typically begins in infancy and progresses in an ascending manner, starting with leg weakness and eventually affecting the upper limbs. Clinically, IAHSP presents with spasticity and hyperreflexia, with muscle stiffness and weakness that worsens over time [157]. The progression of the disease is generally slower compared to ALS and predominantly impacts mobility, without significantly affecting respiratory function or leading to muscle atrophy. While the lower limbs are usually affected first, the involvement of the upper limbs occurs as the disease advances.

Unlike other motor neuron diseases, like ALS, which involves both upper and lower motor neurons, IAHSP predominantly affects upper motor neurons, similarly to PLS. The more aggressive progression of IAHSP compared to PLS and juvenile-onset ALS highlights how the timing and specific nature of Alsin dysfunction can dictate the severity of motor neuron degeneration. IAHSP has been strongly associated with mutations in the *ALS2* gene, which encodes Alsin, a protein involved in intracellular processes like endosomal trafficking and autophagy [158]. Mutations in *ALS2* impair Alsin function, disrupting cellular mechanisms essential for maintaining neuronal health [71]. This leads to the degeneration of motor neurons, resulting in progressive spasticity and muscle weakness.

### 2.5. HSP

Hereditary spastic paraplegia (HSP) is a group of neurodegenerative disorders characterized by progressive spasticity and weakness in the lower limbs due to upper motor neuron degeneration, particularly in the corticospinal tracts [159]. Over 80 specific chromosomal loci associated with HSP, known as SPG loci, have been identified, with the most prevalent and distinct genetic features found in the *SPG4* and *SPG7* genes [160].

The *SPG4* gene encodes the microtubule-severing protein spastin, which is crucial for maintaining axonal integrity [161]. Mutations in *SPG4* disrupt microtubule dynamics, impairing axonal transport and contributing to axonal degeneration [162]. Such mutations primarily affect intracellular transport mechanisms vital for distributing organelles, proteins, and signaling molecules along the axon [163]. This disruption can result in the accumulation of damaged organelles, such as mitochondria and endosomes, provoking neuroinflammation and oxidative stress [164]. Over time, these pathological changes lead to the degeneration of upper motor neurons, clinically manifesting as spasticity and weakness in the lower limbs [165,166]. Another significant gene, *SPG7*, encodes paraplegin, a mitochondrial protein essential for maintaining mitochondrial function [167]. Loss-of-function mutations in *SPG7* compromise mitochondrial maintenance, resulting in energy deficits and increased oxidative stress. Such mutations lead to impaired mitochondrial dynamics, characterized by reduced mitochondrial transport and disrupted fission and fusion processes. This dysfunction causes mitochondrial fragmentation and decreased bioenergetic capacity, which are particularly detrimental to long motor neurons that heavily rely on mitochondrial function for their energy needs. Consequently, these neurons become more susceptible to degeneration, further contributing to the clinical features of HSP, including progressive spasticity and weakness [168,169]. Additionally, while primarily associated with ALS, mutations in the *ALS2* gene have also been implicated in some forms of HSP [170].

### 2.6. PMA

Progressive muscular atrophy (PMA) is a rare neurodegenerative disease that primarily affects lower motor neurons in the spinal cord, leading to progressive muscle weakness and atrophy [171]. Unlike ALS, PMA spares upper motor neurons, resulting in a slower disease progression and longer survival [172]. Despite its clinical overlap with ALS, PMA follows a distinct pathophysiological course.

The genetic basis of PMA is not as well defined as ALS, but some cases have been linked to mutations in *SOD1*, *FUS*, and *TARDBP* [173]. These mutations contribute to neurodegeneration through mechanisms that impair cellular homeostasis. Mutations in *SOD1* lead to oxidative stress and mitochondrial dysfunction, resulting in an accumulation of reactive oxygen species (ROS) that damages motor neurons [18]. Similarly, mutations in *FUS* and *TARDBP* disrupt RNA metabolism, leading to the mislocalization and aggregation of these proteins in the cytoplasm. These aggregates interfere with normal cellular function, further exacerbating motor neuron degeneration [174]. The degeneration of lower motor neurons in PMA causes a progressive loss of muscle innervation, leading to muscle atrophy and weakness. Oxidative stress, mitochondrial dysfunction, and toxic protein aggregation collectively disrupt key cellular pathways necessary for motor neuron survival. As motor neurons deteriorate, muscle weakness becomes more pronounced, ultimately impairing movement and reducing quality of life.

### 2.7. SBMA

Spinal and bulbar muscular atrophy (SBMA), commonly referred to as Kennedy’s disease, is an X-linked recessive disorder resulting from a trinucleotide repeat expansion in the androgen receptor (AR) gene [175]. The mutation associated with SBMA involves an expanded CAG trinucleotide repeat located in exon 1 of the *AR* gene, leading to the toxic accumulation of mutant androgen receptor proteins and progressive degeneration of motor neurons in the spinal cord and bulbar regions [176]. Clinically, SBMA is characterized by muscle weakness and atrophy, causing difficulties in speaking, swallowing, and breathing. SBMA predominantly affects males due to its X-linked inheritance. The disease progresses slowly; however, the loss of motor function eventually leads to significant disability [177].

The genetic basis of SBMA is rooted in the CAG trinucleotide repeat expansion in the *AR* gene, resulting in an elongated polyglutamine (polyQ) tract within the *AR* protein, leading to its toxic gain of function [178]. The pathogenic mechanism begins with the ligand androgen-dependent nuclear accumulation of the mutant AR protein, which initiates detrimental cellular events, including transcriptional dysregulation, where the mutant *AR* alters the expression of genes critical for neuronal survival and function. This disruption negatively impacts the synthesis of essential proteins needed for maintaining motor neuron integrity. Furthermore, the presence of the mutant *AR* protein disrupts axonal transport, vital for distributing organelles, proteins, and signaling molecules along the axon, leading to the accumulation of damaged organelles, contributing to neuronal stress and degeneration [179,180]. Additionally, the mutant *AR* induces mitochondrial dysfunction, characterized by compromised energy production and increased oxidative stress, which is particularly detrimental to lower motor neurons that have high energy demands for maintaining their long axonal projections [181,182]. Collectively, these molecular alterations driven by the genetic mutation culminate in the degeneration of lower motor neurons in the spinal cord and brainstem, resulting in the clinical manifestations of SBMA.

### 2.8. LCCS

Lethal congenital contracture syndrome (LCCS) is characterized by multiple congenital contractures, severe muscle weakness, and significant developmental abnormalities [183]. Affected individuals typically present with joint contractures at birth that restrict movement, alongside profound muscle weakness that impedes voluntary movements and delays motor milestones. Respiratory complications are common due to weakened respiratory muscles, leading to a high risk of respiratory failure, often necessitating mechanical ventilation. Additionally, skeletal abnormalities, such as scoliosis, may complicate the clinical picture. As a result of these manifestations, LCCS usually leads to a fatal outcome in infancy or early childhood [184]. The genetic variability associated with LCCS contributes to a spectrum of clinical manifestations, with distinct types exhibiting unique features and severity. This highlights the importance of precise genetic testing and counseling for affected families.

LCCS is divided into three categories—LCCS1, LCCS2, and LCCS3— based on distinct genetic mutations that disrupt specific cellular signaling pathways, ultimately leading to motor neuron dysfunction and degeneration. Each category exhibits unique clinical features and mechanisms, showcasing the syndrome’s complexity. The underlying pathophysiology comprises a mix of myopathic and neurogenic factors that contribute to its clinical manifestations. LCCS1 is an autosomal recessive condition, presenting with fetal akinesia and targeted degeneration of motor neurons in the spinal cord. The causative mutations in GLE1, which encodes a protein essential for the export of mRNAs from the nucleus to the cytoplasm, disrupt the proper processing and transport of mRNA [185,186]. This leads to an impaired protein synthesis mechanism in motor neurons, contributing to their degeneration. In a significant majority of cases, specifically 51 out of 52, individuals were found to be homozygous for a single substitution in intron 3 (c.432-10A > G). This mutation creates a cryptic splice acceptor site, leading to the insertion of three additional amino acids into the coiled-coil domain of the GLE1 protein. This alteration likely disrupts its interaction with motor neuron-specific proteins, resulting in the selective degeneration of anterior horn motor neurons [187]. LCCS2 is caused by mutations in the *ERBB3* gene. Unlike LCCS1, the pathological changes associated with *ERBB3* mutations are not limited to motor neurons but rather lead to broader neuronal apoptosis. These mutations impair growth factor signaling pathways that regulate neuronal survival, affecting not just motor neurons but various other types of neurons as well. LCCS3 is linked to mutations in the *PIP5K1C* gene, which encodes phosphatidylinositol-4-phosphate 5-kinase type I gamma. Similar to LCCS1, *PIP5K1C* mutations result in the atrophy of the anterior horn of the spinal cord, indicating a targeted degeneration of motor neurons. The role of *PIP5K1C* in maintaining motor neuron integrity highlights its importance in the pathophysiology of LCCS3 [188].

## 3. Epigenetics

### 3.1. Epigenetic Mechanisms in ALS

Epigenetic modifications play a crucial role in ALS pathogenesis by regulating gene expression without altering the DNA sequence. Key mechanisms include DNA methylation, histone modifications, and non-coding RNA dysregulation, all of which contribute to disease progression by influencing gene expression and protein aggregation [189]. ALS motor neurons exhibit widespread changes in DNA methylation and hydroxymethylation, affecting genes linked to immune and inflammatory responses. Loss of *Dnmt3a*, a gene coding a key DNA methyltransferase, reduces motor neuron survival and mimics *SOD1*-mutant ALS models [190]. Hydroxymethylation levels also correlate with protein aggregation: neurons with misfolded *SOD1* show increased 5hmC, whereas those with TDP-43 aggregates have reduced levels [191]. 5-Hydroxymethylcytosine (5hmC) is an intermediate of active DNA demethylation that influences gene expression by altering chromatin accessibility, potentially affecting transcriptional regulation in ALS motor neurons [192]. Histone acetylation is disrupted due to dysregulated histone deacetylase (HDAC) activity. HDAC6, which regulates vesicle transport and autophagy, is notably reduced in late-stage ALS, impairing protein clearance [193]. Additionally, *FUS* overexpression leads to hypoacetylation of *CCND1*, disrupting cell cycle regulation. Cyclin D1 (*CCND1*) is a key regulator of the cell cycle that promotes the transition from the G1 to S phase, and its hypoacetylation in ALS may contribute to aberrant cell cycle activation in post-mitotic neurons, a process linked to neuronal dysfunction and death [194]. The histone acetyltransferase ELP3 is also implicated in motor neuron degeneration, suggesting that histone hypoacetylation contributes to ALS onset [195]. Non-coding RNAs, particularly microRNAs (miRNAs) and long non-coding RNAs (lncRNAs), regulate ALS-related pathways. Specific miRNAs, such as miR-27b-3p and miR-181c-5p, influence TDP-43 levels, while lncRNA NEAT1 promotes nuclear paraspeckle formation, affecting protein sequestration [196]. Dysregulation of these networks enhances the mislocalization and aggregation of TDP-43, *FUS*, and other ALS-linked proteins.

Furthermore, miR-27b-3p is upregulated in the cerebrospinal fluid of ALS patients, contributing to impaired PINK1-mediated mitophagy by directly targeting the 3′-UTR of the PINK1 gene, thereby exacerbating mitochondrial dysfunction. MiR-181c-5p has also been implicated in ALS pathogenesis by inhibiting parkin-mediated mitophagy and sensitizing neurons to mitochondrial stress-induced apoptosis. The dysregulation of these miRNAs further highlights the contribution of mitochondrial impairment to ALS progression [197].

### 3.2. Epigenetic Mechanisms in SMA

Epigenetic modifications significantly influence the expression of the *SMN2* gene, which is crucial for compensating for the loss of *SMN1* in SMA patients. Alterations in DNA methylation levels at the *SMN2* promoter region have been associated with changes in gene expression, suggesting that hypomethylation could enhance *SMN2* transcription [198]. Histone modifications, such as acetylation and methylation, influence chromatin structure and accessibility, thereby affecting the recruitment of splicing factors essential for exon 7 inclusion [199]. Specifically, a more relaxed chromatin state, characterized by histone acetylation, has been correlated with increased exon 7 inclusion, leading to higher production of functional SMN protein. A slower RNA polymerase II elongation rate has been associated with increased exon 7 inclusion, as it provides a temporal window that favors the recruitment of splicing enhancers over silencers [200]. This kinetic coupling between transcription and splicing underscores the potential of targeting transcriptional dynamics as a therapeutic strategy [201].

## 4. Current Treatment Approaches of MNDs

The clinical management of MNDs primarily focuses on symptomatic relief and supportive care, as curative treatments remain elusive. Multidisciplinary care approaches have been shown to improve quality of life and, in some cases, extend survival. Despite advancements in understanding the genetic underpinnings of MNDs, translating these discoveries into effective therapies has been challenging. The identification of various genetic mutations associated with conditions like ALS and SMA has provided insights into disease mechanisms. However, the development of targeted treatments that can modify disease progression remains limited. This gap highlights the need for continued research to bridge the divide between genetic findings and clinical applications, aiming to develop therapies that can alter the course of these debilitating diseases.

### 4.1. Current Treatment for ALS

Riluzole, a benzothiazole derivative, was approved by the U.S. Food and Drug Administration (FDA) in 1995 as the first pharmacological treatment for amyotrophic lateral sclerosis (ALS) [202]. It remains a cornerstone in ALS therapy, offering a modest yet meaningful extension of survival. The mechanism of action of riluzole primarily involves the inhibition of glutamatergic neurotransmission, a key pathway implicated in ALS pathogenesis. Excessive glutamate activity in the central nervous system leads to excitotoxicity, a process where overstimulation of neurons by glutamate results in cell death. Riluzole mitigates this by reducing the presynaptic release of glutamate, inactivating voltage-gated sodium channels, and modulating intracellular signaling pathways linked to excitatory amino acid receptors. This reduction in excitotoxic stress helps slow the degeneration of motor neurons, preserving their function for a longer period [203,204].

Riluzole is generally well tolerated, with common adverse effects including nausea, fatigue (asthenia), dizziness, and elevated liver enzymes. Given the potential for hepatotoxicity, regular monitoring of liver function tests is recommended, particularly during the initial months of therapy. Rare but serious side effects such as neutropenia and interstitial lung disease have also been reported, necessitating vigilance during long-term treatment [205]. Despite its limitations, most notably its inability to halt or reverse motor neuron degeneration, riluzole remains an essential component of ALS treatment. Its neuroprotective effects, although limited, contribute to prolonging patient survival and delaying the need for invasive ventilation.

Tofersen is an antisense oligonucleotide (ASO), a short synthetic strand of nucleotides designed to bind selectively to RNA, specifically developed to target SOD1 mutations implicated in certain forms of ALS [206]. By binding to SOD1 mRNA, tofersen facilitates its degradation, thereby reducing the production of the toxic SOD1 protein that contributes to motor neuron degeneration. Clinical studies have demonstrated that tofersen effectively lowers SOD1 protein levels in the cerebrospinal fluid, offering a targeted therapeutic approach for individuals with SOD1-associated ALS [207].

### 4.2. Current Treatment for SMA

The current treatments for SMA include Spinraza, Zolgensma, and Evrysdi. Spinraza^®^ is an antisense oligonucleotide administered intrathecally for the treatment of SMA in pediatric and adult patients. The recommended dosage is 12 mg per administration, initiated with four loading doses: the first three at 14-day intervals, the fourth 30 days after the third, followed by maintenance doses every four months. Nusinersen enhances the production of functional SMN protein by modifying *SMN2* pre-mRNA splicing, promoting exon 7 inclusion, which is crucial for motor neuron survival. Common adverse effects include headache, back pain, and post-lumbar puncture syndrome. Limitations involve the need for repeated intrathecal administrations and monitoring for potential adverse reactions associated with lumbar puncture [208]. Zolgensma^®^ is a one-time gene therapy delivered via intravenous infusion, indicated for pediatric patients less than 2 years old with SMA. The recommended dose is 1.1 × 10^14^ vector genomes per kilogram of body weight.

This therapy utilizes an adeno-associated virus serotype 9 (AAV9) vector to deliver a functional copy of the *SMN1* gene, leading to sustained SMN protein expression and improved motor function. Common adverse reactions include elevated liver enzymes and vomiting; serious liver injury and acute liver failure have been reported, necessitating liver function monitoring. Limitations include its high cost and the need for pre- and post-treatment with corticosteroids to manage potential hepatotoxicity [209]. Evrysdi^®^ is an orally administered small-molecule therapy for daily use in patients with SMA, aged 2 months and older. It functions as an *SMN2* splicing modifier, increasing the production of functional SMN protein systemically by promoting exon 7 inclusion during *SMN2* pre-mRNA splicing. Common adverse effects include fever, diarrhea, and rash. Limitations involve the necessity for daily lifelong administration and potential drug interactions due to its systemic nature [210]. Recent research has investigated the potential benefits of prenatal therapy for SMA. In a groundbreaking case, risdiplam was administered to a fetus diagnosed with SMA at 32 weeks of gestation. Now over two years old, the child shows no evident symptoms of the disease, indicating that early intervention could lead to significantly improved outcomes [211].

### 4.3. Current Treatment for Other MNDs

Regarding PLS, although no disease-modifying treatments are currently available, research is exploring gene therapy approaches to address *ALS2* dysfunction. These strategies aim to restore normal Alsin function, potentially slowing or halting disease progression. However, such therapies are still in the preclinical stage, and further studies are needed to assess their safety and efficacy [212]. Current treatments for HSP focus on symptom management through physical therapy and medications like baclofen or tizanidine to reduce spasticity. Experimental therapies are investigating the modulation of mitochondrial function, especially in *SPG7*-related HSP [213,214]. Regarding SBMA, approaches include the use of antisense oligonucleotides to reduce mutant AR expression. While these strategies have shown promise in preclinical models, clinical trials are necessary to determine their therapeutic potential in humans [215].

### 4.4. Potential Treatment for IAHSP

Therapeutic strategies for IAHSP primarily focus on addressing *ALS2* dysfunction to prevent motor neuron degeneration. Gene therapy approaches aim to restore Alsin function, while small-molecule modulators and neuroprotective agents are being explored to enhance intracellular trafficking and autophagy. Although these treatments remain in the experimental stage, ongoing research seeks to evaluate their potential for clinical application [216].

## 5. From Molecular Landscape to Therapy: Future Perspectives in MNDs

Recently, antisense oligonucleotides (ASOs) have emerged as a promising strategy, particularly in genetically linked forms of ALS. The success of the SOD1Rx trial has demonstrated the potential of ASOs in modifying disease progression, paving the way for their application in *C9orf72*-related ALS. Studies have shown that ASOs targeting *C9orf72* can effectively reduce toxic RNA species, decrease neuronal dysfunction, and suppress pathological RNA aggregation in patient-derived neurons. Preclinical models further support their therapeutic viability, with a single intracerebroventricular ASO injection leading to prolonged suppression of pathological transcripts without adverse effects. These findings reinforce ASOs as a viable long-term therapeutic approach for *C9orf72*-related ALS [217].

Future therapeutic strategies for MNDs focus on innovative, targeted approaches that address the underlying genetic mutations. Gene therapy and advances in gene editing technologies hold promise as developmental therapies for ALS and SMA by aiming to correct mutations at the DNA level in genes like *C9orf72*, *SOD1*, *TARDBP*, *ATXN2,* and *SMN1*. ASOs can potentially modify RNA processing and reduce toxic protein buildup, with emerging applications in SMA [206]. Additionally, small-molecule modulators are being explored to enhance autophagy and combat oxidative stress, protecting neurons from mutation-related damage [218]. Advances in precision medicine may lead to strategies that enable treatments tailored to each patient’s genetic profile, increasing efficacy and reducing side effects. Early biomarker identification and genetic testing could also allow for more proactive interventions. Emerging insights into the role of non-neuronal cells, like glial cells, may lead to supportive therapies targeting the broader neural environment. As our understanding of MND pathways advances, the potential for combination therapies grows, offering renewed hope for effective disease management and improved quality of life.

Due to its role in enhancing cellular defense against oxidative stress, the Nrf2/ARE pathway is considered a promising therapeutic target for MNDs. Pharmacological activators, like dimethyl fumarate, have shown potential in delaying disease progression and reducing neuroinflammation, offering hope for extending the lifespan of ALS patients [219,220]. Research into the therapeutic potential of targeting *FUS* dysfunction is ongoing, with several strategies being explored to prevent mislocalization and aggregation of the *FUS* protein. ASOs have shown promise in reducing *FUS* levels and mitigating disease progression in preclinical models. These therapeutic strategies hold potential for treating *FUS*-related ALS [221]. All the aforementioned techniques can potentially be employed in the genes that have been analyzed in this review.

## 6. Conclusions

MNDs are complex neurodegenerative disorders caused by genetic mutations that disrupt essential cellular processes. Mutations in genes like *C9orf72, SOD1, TARDBP*, and *FUS* play a significant role in ALS, through mechanisms such as oxidative stress, RNA metabolism impairment, and protein misfolding. In SMA, *SMN1* mutations drive progressive motor neuron loss and muscle weakness. In HSP, mutations in *SPG4* and SPG7 impact axonal transport and mitochondrial function, while *ALS2* mutations are associated with juvenile ALS, PLS, and certain HSP forms, affecting autophagy and vesicle trafficking. Expanding our understanding of these mutations has revealed additional pathogenic mechanisms that could be potentially targeted. *C9orf72* repeat expansions disrupt nucleocytoplasmic transport and form toxic RNA foci, leading to widespread cellular dysfunction. *SOD1* mutations promote misfolded protein aggregation, increasing oxidative stress and mitochondrial damage. *TARDBP* and *FUS* mutations impair RNA metabolism and stress granule dynamics, driving toxic protein aggregation and neuronal dysfunction. In *SMA*, reduced SMN protein disrupts snRNP assembly, compromising pre-mRNA splicing and causing RNA instability, particularly in motor neurons. In HSP, *SPG4* mutations impair microtubule severing, while *SPG7* mutations lead to mitochondrial dysfunction. These insights highlight the diverse molecular pathways driving MNDs and emphasize the need for innovative targeted therapies. Precision medicine therapies are at the epicenter of treating MNDs and improving patient outcomes.

## Figures and Tables

**Table 1:** Genes related to ALS pathogenesis.

Gene Name	Encoded Protein	Chromosomal Locus	Pathogenic Mechanism	Ref.
*SOD1*	Superoxide dismutase 1	21q22.11	Mutant SOD1 misfolds, aggregates, and induces oxidative stress, mitochondrial dysfunction, and motor neuron death.	[39,46]
*TARDBP*	TAR DNA-binding protein 43	1p36.22	TDP-43 mislocalizes, forming toxic aggregates that disrupt RNA processing, autophagy, and neuronal homeostasis.	[62]
*FUS*	Fused in sarcoma	16p11.2	Cytoplasmic mislocalization causes toxic aggregation, impaired RNA metabolism, and stress granule dysfunction.	[68]
*C9orf72*	C9orf72 protein	9p21.2	G4C2 repeat expansion forms RNA foci, toxic dipeptides, disrupts autophagy, and induces neurotoxicity.	[69,70]
*ALS2*	Alsin	2q33.1	Loss of function disrupts endosomal trafficking, autophagy, and intracellular transport, causing motor neuron degeneration.	[71]
*ATXN2*	Ataxin-2	12q24.12	PolyQ expansions enhance TDP-43 aggregation, impair RNA metabolism, and increase ALS risk.	[72]

Information on the full names of genes and chromosomal loci was obtained from The Human Protein Atlas (https://www.proteinatlas.org). ALS2: Alsin; ATXN2: Ataxin-2; C9orf72: Chromosome 9 Open Reading Frame 72; FUS: Fused in Sarcoma; SOD1: Superoxide Dismutase 1; TDP-43: TAR DNA-binding protein 43.

## Data Availability

Not applicable.

## Abbreviations

The following abbreviations are used in this manuscript:

ALS	Amyotrophic Lateral Sclerosis
ALS-FTD	Amyotrophic Lateral Sclerosis–Frontotemporal Dementia
ALS2	Amyotrophic Lateral Sclerosis 2
ANG	Angiogenin
AR	Androgen Receptor
ASO	Antisense Oligonucleotide
ATL1	Atlastin GTPase 1
ATP	Adenosine Triphosphate
ATXN2	Ataxin-2
BBB	Blood–Brain Barrier
C9orf72	Chromosome 9 Open Reading Frame 72
CAG	Cytosine–Adenine–Guanine
CCND1	Cyclin D1
CHIP	Carboxyl Terminus of Hsc70-Interacting Protein
CHMP2B	Charged Multivesicular Body Protein 2B
CNS	Central Nervous System
DAPI	4′,6-Diamidino-2-Phenylindole
DOAJ	Directory of Open Access Journals
DPR	Dipeptide Repeat Proteins
ELISA	Enzyme-Linked Immunosorbent Assay
ERBB3	Erb-B2 Receptor Tyrosine Kinase 3
FBS	Fetal Bovine Serum
FIG4	FIG4 Phosphoinositide 5-Phosphatase
FTD	Frontotemporal Dementia
FUS	Fused in Sarcoma
GFAP	Glial Fibrillary Acidic Protein
GLE1	GLE1 RNA Export Mediator
GRN	Progranulin
HDAC	Histone Deacetylase
HSP	Hereditary Spastic Paraplegia
HSP70	Heat Shock Protein 70
IAHSP	Infantile-Onset Ascending Hereditary Spastic Paraplegia
KIF5A	Kinesin Family Member 5A
LCCS	Lethal Congenital Contracture Syndrome
LD	Linear Dichroism
MAPT	Microtubule-Associated Protein Tau
MDPI	Multidisciplinary Digital Publishing Institute
MND	Motor Neuron Disease
mRNA	Messenger Ribonucleic Acid
NES	Nuclear Export Signal
NMJ	Neuromuscular Junction
Nrf2/ARE	Nuclear Factor Erythroid 2-Related Factor 2/Antioxidant Response Element
OPTN	Optineurin
PBS	Phosphate-Buffered Saline
PIP5K1C	Phosphatidylinositol-4-Phosphate 5-Kinase Type I Gamma
PLS	Primary Lateral Sclerosis
PMA	Progressive Muscular Atrophy
PolyQ	Polyglutamine
REEP1	Receptor Expression-Enhancing Protein 1
RNA	Ribonucleic Acid
RNP	Ribonucleoprotein
ROS	Reactive Oxygen Species
RT-PCR	Reverse Transcription Polymerase Chain Reaction
Rab5	Ras-related Protein Rab-5
SBMA	Spinal and Bulbar Muscular Atrophy
siRNA	Small Interfering RNA
SMA	Spinal Muscular Atrophy
SMN	Survival Motor Neuron
SOD1	Superoxide Dismutase 1
SPAST	Spastin
SPG4	Spastin
SPG7	Paraplegin
TARDBP	TAR DNA-Binding Protein Gene
TDP-43	TAR DNA-Binding Protein 43
TFEB	Transcription Factor EB
TUBA4A	Tubulin Alpha 4A
UBQLN2	Ubiquilin-2
UPR	Unfolded Protein Response
UPS	Ubiquitin–Proteasome System
VCP	Valosin-Containing Protein
5hmC	5-hydroxymethylcytosine

## References

[1] Foster L.A., Salajegheh M.K. (2019). Motor Neuron Disease: Pathophysiology, Diagnosis, and Management. Am. J. Med..

[2] Radakovic R., Radakovic C., Abrahams S., Simmons Z., Carroll A. (2024). Quality of Life, Cognitive and Behavioural Impairment in People with Motor Neuron Disease: A Systematic Review. Qual. Life Res..

[3] Quinn C., Elman L. (2020). Amyotrophic Lateral Sclerosis and Other Motor Neuron Diseases. Continuum Lifelong Learn. Neurol..

[4] Cookson M.R., Shaw P.J. (1999). Oxidative Stress and Motor Neurone Disease. Brain Pathol..

[5] Xu J., Su X., Burley S.K., Zheng X.F.S. (2022). Nuclear SOD1 in Growth Control, Oxidative Stress Response, Amyotrophic Lateral Sclerosis, and Cancer. Antioxidants.

[6] Brotman R.G., Moreno-Escobar M.C., Joseph J., Munakomi S., Pawar G. (2024). Amyotrophic Lateral Sclerosis. StatPearls.

[7] Masrori P., Van Damme P. (2020). Amyotrophic Lateral Sclerosis: A Clinical Review. Eur. J. Neurol..

[8] Verma A., Araki T. (2021). Clinical Manifestation and Management of Amyotrophic Lateral Sclerosis. Amyotrophic Lateral Sclerosis.

[9] Zakharova M.N., Abramova A.A. (2022). Lower and Upper Motor Neuron Involvement and Their Impact on Disease Prognosis in Amyotrophic Lateral Sclerosis. Neural Regen. Res..

[10] Tena A., Clarià F., Solsona F., Povedano M. (2022). Detecting Bulbar Involvement in Patients with Amyotrophic Lateral Sclerosis Based on Phonatory and Time-Frequency Features. Sensors.

[11] Corcia P., Pradat P.-F., Salachas F., Bruneteau G., Forestier N.L., Seilhean D., Hauw J.-J., Meininger V. (2008). Causes of Death in a Post-Mortem Series of ALS Patients. Amyotroph. Lateral Scler..

[12] Mathis S., Goizet C., Soulages A., Vallat J.-M., Masson G.L. (2019). Genetics of Amyotrophic Lateral Sclerosis: A Review. J. Neurol. Sci..

[13] Ticozzi N., Tiloca C., Morelli C., Colombrita C., Poletti B., Doretti A., Maderna L., Messina S., Ratti A., Silani V. (2011). Genetics of Familial Amyotrophic Lateral Sclerosis. Arch. Ital. Biol..

[14] Gibson S.B., Downie J.M., Tsetsou S., Feusier J.E., Figueroa K.P., Bromberg M.B., Jorde L.B., Pulst S.M. (2017). The Evolving Genetic Risk for Sporadic ALS. Neurology.

[15] Hemerková P., Vališ M. (2021). Role of Oxidative Stress in the Pathogenesis of Amyotrophic Lateral Sclerosis: Antioxidant Metalloenzymes and Therapeutic Strategies. Biomolecules.

[16] Barber S.C., Mead R.J., Shaw P.J. (2006). Oxidative Stress in ALS: A Mechanism of Neurodegeneration and a Therapeutic Target. Biochim. Biophys. Acta BBA Mol. Basis Dis..

[17] Pizzino G., Irrera N., Cucinotta M., Pallio G., Mannino F., Arcoraci V., Squadrito F., Altavilla D., Bitto A. (2017). Oxidative Stress: Harms and Benefits for Human Health. Oxid. Med. Cell Longev..

[18] Chaudhary P., Janmeda P., Docea A.O., Yeskaliyeva B., Abdull Razis A.F., Modu B., Calina D., Sharifi-Rad J. (2023). Oxidative Stress, Free Radicals and Antioxidants: Potential Crosstalk in the Pathophysiology of Human Diseases. Front. Chem..

[19] Zelko I.N., Mariani T.J., Folz R.J. (2002). Superoxide Dismutase Multigene Family: A Comparison of the CuZn-SOD (SOD1), Mn-SOD (SOD2), and EC-SOD (SOD3) Gene Structures, Evolution, and Expression. Free. Radic. Biol. Med..

[20] Tsang C.K., Liu Y., Thomas J., Zhang Y., Zheng X.F.S. (2014). Superoxide Dismutase 1 Acts as a Nuclear Transcription Factor to Regulate Oxidative Stress Resistance. Nat. Commun..

[21] Li Y., Wang T., Li X., Li W., Lei Y., Shang Q., Zheng Z., Fang J., Cao L., Yu D. (2024). SOD2 Promotes the Immunosuppressive Function of Mesenchymal Stem Cells at the Expense of Adipocyte Differentiation. Mol. Ther..

[22] Parascandolo A., Laukkanen M.O. (2021). SOD3 Is a Non-Mutagenic Growth Regulator Affecting Cell Migration and Proliferation Signal Transduction. Antioxidants.

[23] Zgorzynska E., Dziedzic B., Walczewska A. (2021). An Overview of the Nrf2/ARE Pathway and Its Role in Neurodegenerative Diseases. Int. J. Mol. Sci..

[24] Petri S., Körner S., Kiaei M. (2012). Nrf2/ARE Signaling Pathway: Key Mediator in Oxidative Stress and Potential Therapeutic Target in ALS. Neurol. Res. Int..

[25] Li L., Dong H., Song E., Xu X., Liu L., Song Y. (2014). Nrf2/ARE Pathway Activation, HO-1 and NQO1 Induction by Polychlorinated Biphenyl Quinone Is Associated with Reactive Oxygen Species and PI3K/AKT Signaling. Chem. Biol. Interact..

[26] Harvey C.J., Thimmulappa R.K., Singh A., Blake D.J., Ling G., Wakabayashi N., Fujii J., Myers A., Biswal S. (2008). Nrf2-Regulated Glutathione Recycling Independent of Biosynthesis Is Critical for Cell Survival during Oxidative Stress. Free. Radic. Biol. Med..

[27] Canella R., Benedusi M., Martini M., Cervellati F., Cavicchio C., Valacchi G. (2018). Role of Nrf2 in Preventing Oxidative Stress Induced Chloride Current Alteration in Human Lung Cells. J. Cell Physiol..

[28] Nguyen T., Nioi P., Pickett C.B. (2009). The Nrf2-Antioxidant Response Element Signaling Pathway and Its Activation by Oxidative Stress. J. Biol. Chem..

[29] Goodfellow M.J., Borcar A., Proctor J.L., Greco T., Rosenthal R.E., Fiskum G. (2020). Transcriptional Activation of Antioxidant Gene Expression by Nrf2 Protects against Mitochondrial Dysfunction and Neuronal Death Associated with Acute and Chronic Neurodegeneration. Exp. Neurol..

[30] Vomund S., Schäfer A., Parnham M.J., Brüne B., von Knethen A. (2017). Nrf2, the Master Regulator of Anti-Oxidative Responses. Int. J. Mol. Sci..

[31] Bono S., Feligioni M., Corbo M. (2021). Impaired Antioxidant KEAP1-NRF2 System in Amyotrophic Lateral Sclerosis: NRF2 Activation as a Potential Therapeutic Strategy. Mol. Neurodegener..

[32] Coque E., Salsac C., Espinosa-Carrasco G., Varga B., Degauque N., Cadoux M., Crabé R., Virenque A., Soulard C., Fierle J.K. (2019). Cytotoxic CD8+ T Lymphocytes Expressing ALS-Causing SOD1 Mutant Selectively Trigger Death of Spinal Motoneurons. Proc. Natl. Acad. Sci. USA.

[33] Mead R.J., Bennett E.J., Kennerley A.J., Sharp P., Sunyach C., Kasher P., Berwick J., Pettmann B., Battaglia G., Azzouz M. (2011). Optimised and Rapid Pre-Clinical Screening in the SOD1(G93A) Transgenic Mouse Model of Amyotrophic Lateral Sclerosis (ALS). PLoS ONE.

[34] Saeed M., Yang Y., Deng H.-X., Hung W.-Y., Siddique N., Dellefave L., Gellera C., Andersen P.M., Siddique T. (2009). Age and Founder Effect of SOD1 A4V Mutation Causing ALS. Neurology.

[35] Singh A., Kukreti R., Saso L., Kukreti S. (2019). Oxidative Stress: A Key Modulator in Neurodegenerative Diseases. Molecules.

[36] Arslanbaeva L., Bisaglia M. (2022). Activation of the Nrf2 Pathway as a Therapeutic Strategy for ALS Treatment. Molecules.

[37] Soo K.Y., Atkin J.D., Horne M.K., Nagley P. (2009). Recruitment of Mitochondria into Apoptotic Signaling Correlates with the Presence of Inclusions Formed by Amyotrophic Lateral Sclerosis-Associated SOD1 Mutations. J. Neurochem..

[38] Cozzolino M., Pesaresi M.G., Amori I., Crosio C., Ferri A., Nencini M., Carrì M.T. (2009). Oligomerization of Mutant SOD1 in Mitochondria of Motoneuronal Cells Drives Mitochondrial Damage and Cell Toxicity. Antioxid. Redox Signal.

[39] Tafuri F., Ronchi D., Magri F., Comi G.P., Corti S. (2015). SOD1 Misplacing and Mitochondrial Dysfunction in Amyotrophic Lateral Sclerosis Pathogenesis. Front. Cell Neurosci..

[40] Li Q., Spencer N.Y., Pantazis N.J., Engelhardt J.F. (2011). Alsin and SOD1(G93A) Proteins Regulate Endosomal Reactive Oxygen Species Production by Glial Cells and Proinflammatory Pathways Responsible for Neurotoxicity. J. Biol. Chem..

[41] Meissner F., Molawi K., Zychlinsky A. (2010). Mutant Superoxide Dismutase 1-Induced IL-1β Accelerates ALS Pathogenesis. Proc. Natl. Acad. Sci. USA.

[42] Dermitzakis I., Manthou M.E., Meditskou S., Tremblay M.-È., Petratos S., Zoupi L., Boziki M., Kesidou E., Simeonidou C., Theotokis P. (2023). Origin and Emergence of Microglia in the CNS—An Interesting (Hi)Story of an Eccentric Cell. CIMB.

[43] Ferri A., Nencini M., Casciati A., Cozzolino M., Angelini D.F., Longone P., Spalloni A., Rotilio G., Carrì M.T. (2004). Cell Death in Amyotrophic Lateral Sclerosis: Interplay between Neuronal and Glial Cells. FASEB J..

[44] Nagai M., Re D.B., Nagata T., Chalazonitis A., Jessell T.M., Wichterle H., Przedborski S. (2007). Astrocytes Expressing ALS-Linked Mutated SOD1 Release Factors Selectively Toxic to Motor Neurons. Nat. Neurosci..

[45] Geloso M.C., Corvino V., Marchese E., Serrano A., Michetti F., D’Ambrosi N. (2017). The Dual Role of Microglia in ALS: Mechanisms and Therapeutic Approaches. Front. Aging Neurosci..

[46] Peggion C., Scalcon V., Massimino M.L., Nies K., Lopreiato R., Rigobello M.P., Bertoli A. (2022). SOD1 in ALS: Taking Stock in Pathogenic Mechanisms and the Role of Glial and Muscle Cells. Antioxidants.

[47] Colombrita C., Onesto E., Tiloca C., Ticozzi N., Silani V., Ratti A. (2011). RNA-Binding Proteins and RNA Metabolism: A New Scenario in the Pathogenesis of Amyotrophic Lateral Sclerosis. Arch. Ital. Biol..

[48] Cohen T.J., Lee V.M.Y., Trojanowski J.Q. (2011). TDP-43 Functions and Pathogenic Mechanisms Implicated in TDP-43 Proteinopathies. Trends Mol. Med..

[49] Tziortzouda P., Van Den Bosch L., Hirth F. (2021). Triad of TDP43 Control in Neurodegeneration: Autoregulation, Localization and Aggregation. Nat. Rev. Neurosci..

[50] Birsa N., Bentham M.P., Fratta P. (2020). Cytoplasmic Functions of TDP-43 and FUS and Their Role in ALS. Semin. Cell Dev. Biol..

[51] Nagano S., Jinno J., Abdelhamid R.F., Jin Y., Shibata M., Watanabe S., Hirokawa S., Nishizawa M., Sakimura K., Onodera O. (2020). TDP-43 Transports Ribosomal Protein mRNA to Regulate Axonal Local Translation in Neuronal Axons. Acta Neuropathol..

[52] Wong C.-E., Jin L.-W., Chu Y.-P., Wei W.-Y., Ho P.-C., Tsai K.-J. (2021). TDP-43 Proteinopathy Impairs mRNP Granule Mediated Postsynaptic Translation and mRNA Metabolism. Theranostics.

[53] Mori F., Yasui H., Miki Y., Kon T., Arai A., Kurotaki H., Tomiyama M., Wakabayashi K. (2024). Colocalization of TDP-43 and Stress Granules at the Early Stage of TDP-43 Aggregation in Amyotrophic Lateral Sclerosis. Brain Pathol..

[54] Dewey C.M., Cenik B., Sephton C.F., Johnson B.A., Herz J., Yu G. (2012). TDP-43 Aggregation in Neurodegeneration: Are Stress Granules the Key?. Brain Res..

[55] Rutherford N.J., Zhang Y.-J., Baker M., Gass J.M., Finch N.A., Xu Y.-F., Stewart H., Kelley B.J., Kuntz K., Crook R.J.P. (2008). Novel Mutations in TARDBP (TDP-43) in Patients with Familial Amyotrophic Lateral Sclerosis. PLoS Genet..

[56] Sreedharan J., Blair I.P., Tripathi V.B., Hu X., Vance C., Rogelj B., Ackerley S., Durnall J.C., Williams K.L., Buratti E. (2008). TDP-43 Mutations in Familial and Sporadic Amyotrophic Lateral Sclerosis. Science.

[57] Janssens J., Wils H., Kleinberger G., Joris G., Cuijt I., Ceuterick-de Groote C., Van Broeckhoven C., Kumar-Singh S. (2013). Overexpression of ALS-Associated p.M337V Human TDP-43 in Mice Worsens Disease Features Compared to Wild-Type Human TDP-43 Mice. Mol. Neurobiol..

[58] Zeng J., Tang Y., Dong X., Li F., Wei G. (2024). Influence of ALS-Linked M337V Mutation on the Conformational Ensembles of TDP-43321-340 Peptide Monomer and Dimer. Proteins.

[59] Takeda T., Iijima M., Shimizu Y., Yoshizawa H., Miyashiro M., Onizuka H., Yamamoto T., Nishiyama A., Suzuki N., Aoki M. (2019). P.N345K Mutation in TARDBP in a Patient with Familial Amyotrophic Lateral Sclerosis: An Autopsy Case. Neuropathology.

[60] Gendron T.F., Rademakers R., Petrucelli L. (2013). TARDBP Mutation Analysis in TDP-43 Proteinopathies and Deciphering the Toxicity of Mutant TDP-43. J. Alzheimers Dis..

[61] Zou Z.-Y., Zhou Z.-R., Che C.-H., Liu C.-Y., He R.-L., Huang H.-P. (2017). Genetic Epidemiology of Amyotrophic Lateral Sclerosis: A Systematic Review and Meta-Analysis. J. Neurol. Neurosurg. Psychiatry.

[62] Suk T.R., Rousseaux M.W.C. (2020). The Role of TDP-43 Mislocalization in Amyotrophic Lateral Sclerosis. Mol. Neurodegener..

[63] De Marco G., Lupino E., Calvo A., Moglia C., Buccinnà B., Grifoni S., Ramondetti C., Lomartire A., Rinaudo M.T., Piccinini M. (2011). Cytoplasmic Accumulation of TDP-43 in Circulating Lymphomonocytes of ALS Patients with and without TARDBP Mutations. Acta Neuropathol..

[64] Barmada S.J., Skibinski G., Korb E., Rao E.J., Wu J.Y., Finkbeiner S. (2010). Cytoplasmic Mislocalization of TDP-43 Is Toxic to Neurons and Enhanced by a Mutation Associated with Familial Amyotrophic Lateral Sclerosis. J. Neurosci..

[65] Wood A., Gurfinkel Y., Polain N., Lamont W., Lyn Rea S. (2021). Molecular Mechanisms Underlying TDP-43 Pathology in Cellular and Animal Models of ALS and FTLD. Int. J. Mol. Sci..

[66] Pakravan D., Michiels E., Bratek-Skicki A., De Decker M., Van Lindt J., Alsteens D., Derclaye S., Van Damme P., Schymkowitz J., Rousseau F. (2021). Liquid-Liquid Phase Separation Enhances TDP-43 LCD Aggregation but Delays Seeded Aggregation. Biomolecules.

[67] Carey J.L., Guo L. (2022). Liquid-Liquid Phase Separation of TDP-43 and FUS in Physiology and Pathology of Neurodegenerative Diseases. Front. Mol. Biosci..

[68] Xiao X., Li M., Ye Z., He X., Wei J., Zha Y. (2024). FUS Gene Mutation in Amyotrophic Lateral Sclerosis: A New Case Report and Systematic Review. Amyotroph. Lateral Scler. Front. Degener..

[69] Gijselinck I., Cruts M., Van Broeckhoven C. (2018). The Genetics of C9orf72 Expansions. Cold Spring Harb. Perspect. Med..

[70] Freibaum B.D., Lu Y., Lopez-Gonzalez R., Kim N.C., Almeida S., Lee K.-H., Badders N., Valentine M., Miller B.L., Wong P.C. (2015). GGGGCC Repeat Expansion in C9orf72 Compromises Nucleocytoplasmic Transport. Nature.

[71] Orrell R.W. (2021). ALS2-Related Disorder. GeneReviews^®^ [Internet].

[72] Elden A.C., Kim H.-J., Hart M.P., Chen-Plotkin A.S., Johnson B.S., Fang X., Armakola M., Geser F., Greene R., Lu M.M. (2010). Ataxin-2 Intermediate-Length Polyglutamine Expansions Are Associated with Increased Risk for ALS. Nature.

[73] Honda D., Ishigaki S., Iguchi Y., Fujioka Y., Udagawa T., Masuda A., Ohno K., Katsuno M., Sobue G. (2014). The ALS/FTLD-Related RNA-Binding Proteins TDP-43 and FUS Have Common Downstream RNA Targets in Cortical Neurons. FEBS Open Bio.

[74] Nolan M., Talbot K., Ansorge O. (2016). Pathogenesis of FUS-Associated ALS and FTD: Insights from Rodent Models. Acta Neuropathol. Commun..

[75] Assoni A.F., Foijer F., Zatz M. (2023). Amyotrophic Lateral Sclerosis, FUS and Protein Synthesis Defects. Stem Cell Rev. Rep..

[76] Bowden H.A., Dormann D. (2016). Altered mRNP Granule Dynamics in FTLD Pathogenesis. J. Neurochem..

[77] López-Erauskin J., Tadokoro T., Baughn M.W., Myers B., McAlonis-Downes M., Chillon-Marinas C., Asiaban J.N., Artates J., Bui A.T., Vetto A.P. (2018). ALS/FTD-Linked Mutation in FUS Suppresses Intra-Axonal Protein Synthesis and Drives Disease Without Nuclear Loss-of-Function of FUS. Neuron.

[78] Dormann D., Rodde R., Edbauer D., Bentmann E., Fischer I., Hruscha A., Than M.E., Mackenzie I.R.A., Capell A., Schmid B. (2010). ALS-Associated Fused in Sarcoma (FUS) Mutations Disrupt Transportin-Mediated Nuclear Import. EMBO J..

[79] Shelkovnikova T.A., Robinson H.K., Connor-Robson N., Buchman V.L. (2013). Recruitment into Stress Granules Prevents Irreversible Aggregation of FUS Protein Mislocalized to the Cytoplasm. Cell Cycle.

[80] Kwiatkowski T.J., Bosco D.A., Leclerc A.L., Tamrazian E., Vanderburg C.R., Russ C., Davis A., Gilchrist J., Kasarskis E.J., Munsat T. (2009). Mutations in the FUS/TLS Gene on Chromosome 16 Cause Familial Amyotrophic Lateral Sclerosis. Science.

[81] An H., Litscher G., Watanabe N., Wei W., Hashimoto T., Iwatsubo T., Buchman V.L., Shelkovnikova T.A. (2022). ALS-Linked Cytoplasmic FUS Assemblies Are Compositionally Different from Physiological Stress Granules and Sequester hnRNPA3, a Novel Modifier of FUS Toxicity. Neurobiol. Dis..

[82] Barmada S.J. (2015). Linking RNA Dysfunction and Neurodegeneration in Amyotrophic Lateral Sclerosis. Neurotherapeutics.

[83] Scekic-Zahirovic J., Sendscheid O., El Oussini H., Jambeau M., Sun Y., Mersmann S., Wagner M., Dieterlé S., Sinniger J., Dirrig-Grosch S. (2016). Toxic Gain of Function from Mutant FUS Protein Is Crucial to Trigger Cell Autonomous Motor Neuron Loss. EMBO J..

[84] Liu E.Y., Cali C.P., Lee E.B. (2017). RNA Metabolism in Neurodegenerative Disease. Dis. Model. Mech..

[85] Daigle J.G., Lanson N.A., Smith R.B., Casci I., Maltare A., Monaghan J., Nichols C.D., Kryndushkin D., Shewmaker F., Pandey U.B. (2013). RNA-Binding Ability of FUS Regulates Neurodegeneration, Cytoplasmic Mislocalization and Incorporation into Stress Granules Associated with FUS Carrying ALS-Linked Mutations. Hum. Mol. Genet..

[86] Scotter E.L., Chen H.-J., Shaw C.E. (2015). TDP-43 Proteinopathy and ALS: Insights into Disease Mechanisms and Therapeutic Targets. Neurotherapeutics.

[87] Lee J.W., Kang S.-W., Choi W.A. (2021). Clinical Course of Amyotrophic Lateral Sclerosis According to Initial Symptoms: An Analysis of 500 Cases. Yonsei Med. J..

[88] Hayes L.R., Kalab P. (2022). Emerging Therapies and Novel Targets for TDP-43 Proteinopathy in ALS/FTD. Neurotherapeutics.

[89] Yan Y.-P., Xu C.-Y., Gu L.-Y., Zhang B., Shen T., Gao T., Tian J., Pu J.-L., Yin X.-Z., Zhang B.-R. (2020). Genetic Testing of FUS, HTRA2, and TENM4 Genes in Chinese Patients with Essential Tremor. CNS Neurosci. Ther..

[90] O’Rourke J.G., Bogdanik L., Yáñez A., Lall D., Wolf A.J., Muhammad A.K.M.G., Ho R., Carmona S., Vit J.P., Zarrow J. (2016). C9orf72 Is Required for Proper Macrophage and Microglial Function in Mice. Science.

[91] Wang T., Liu H., Itoh K., Oh S., Zhao L., Murata D., Sesaki H., Hartung T., Na C.H., Wang J. (2021). C9orf72 Regulates Energy Homeostasis by Stabilizing Mitochondrial Complex I Assembly. Cell Metab..

[92] Beckers J., Tharkeshwar A.K., Van Damme P. (2021). C9orf72 ALS-FTD: Recent Evidence for Dysregulation of the Autophagy-Lysosome Pathway at Multiple Levels. Autophagy.

[93] Marchi P.M., Marrone L., Brasseur L., Coens A., Webster C.P., Bousset L., Destro M., Smith E.F., Walther C.G., Alfred V. (2022). C9ORF72-Derived Poly-GA DPRs Undergo Endocytic Uptake in iAstrocytes and Spread to Motor Neurons. Life Sci. Alliance.

[94] Pang W., Hu F. (2021). Cellular and Physiological Functions of C9ORF72 and Implications for ALS/FTD. J. Neurochem..

[95] Ratti A., Gumina V., Lenzi P., Bossolasco P., Fulceri F., Volpe C., Bardelli D., Pregnolato F., Maraschi A., Fornai F. (2020). Chronic Stress Induces Formation of Stress Granules and Pathological TDP-43 Aggregates in Human ALS Fibroblasts and iPSC-Motoneurons. Neurobiol. Dis..

[96] Kumar V., Hasan G.M., Hassan M.I. (2017). Unraveling the Role of RNA Mediated Toxicity of C9orf72 Repeats in C9-FTD/ALS. Front. Neurosci..

[97] Maharjan N., Künzli C., Buthey K., Saxena S. (2017). C9ORF72 Regulates Stress Granule Formation and Its Deficiency Impairs Stress Granule Assembly, Hypersensitizing Cells to Stress. Mol. Neurobiol..

[98] Prudencio M., Belzil V.V., Batra R., Ross C.A., Gendron T.F., Pregent L.J., Murray M.E., Overstreet K.K., Piazza-Johnston A.E., Desaro P. (2015). Distinct Brain Transcriptome Profiles in C9orf72-Associated and Sporadic ALS. Nat. Neurosci..

[99] Gendron T.F., Bieniek K.F., Zhang Y.-J., Jansen-West K., Ash P.E.A., Caulfield T., Daughrity L., Dunmore J.H., Castanedes-Casey M., Chew J. (2013). Antisense Transcripts of the Expanded C9ORF72 Hexanucleotide Repeat Form Nuclear RNA Foci and Undergo Repeat-Associated Non-ATG Translation in c9FTD/ALS. Acta Neuropathol..

[100] Boivin M., Pfister V., Gaucherot A., Ruffenach F., Negroni L., Sellier C., Charlet-Berguerand N. (2020). Reduced Autophagy upon C9ORF72 Loss Synergizes with Dipeptide Repeat Protein Toxicity in G4C2 Repeat Expansion Disorders. EMBO J..

[101] Geng Y., Cai Q. (2024). Role of C9orf72 Hexanucleotide Repeat Expansions in ALS/FTD Pathogenesis. Front. Mol. Neurosci..

[102] Nonaka T., Masuda-Suzukake M., Hosokawa M., Shimozawa A., Hirai S., Okado H., Hasegawa M. (2018). C9ORF72 Dipeptide Repeat Poly-GA Inclusions Promote Intracellular Aggregation of Phosphorylated TDP-43. Hum. Mol. Genet..

[103] Hadano S., Kunita R., Otomo A., Suzuki-Utsunomiya K., Ikeda J.-E. (2007). Molecular and Cellular Function of ALS2/Alsin: Implication of Membrane Dynamics in Neuronal Development and Degeneration. Neurochem. Int..

[104] ALS2—An Overview | ScienceDirect Topics. https://www.sciencedirect.com/topics/biochemistry-genetics-and-molecular-biology/als2.

[105] Hsu F., Spannl S., Ferguson C., Hyman A.A., Parton R.G., Zerial M. (2018). Rab5 and Alsin Regulate Stress-Activated Cytoprotective Signaling on Mitochondria. Elife.

[106] Cai Q., Ganesan D. (2022). Regulation of Neuronal Autophagy and the Implications in Neurodegenerative Diseases. Neurobiol. Dis..

[107] Chandran J., Ding J., Cai H. (2007). Alsin and the Molecular Pathways of Amyotrophic Lateral Sclerosis. Mol. Neurobiol..

[108] Lai C., Xie C., Shim H., Chandran J., Howell B.W., Cai H. (2009). Regulation of Endosomal Motility and Degradation by Amyotrophic Lateral Sclerosis 2/Alsin. Mol. Brain.

[109] Otomo A., Hadano S., Okada T., Mizumura H., Kunita R., Nishijima H., Showguchi-Miyata J., Yanagisawa Y., Kohiki E., Suga E. (2003). ALS2, a Novel Guanine Nucleotide Exchange Factor for the Small GTPase Rab5, Is Implicated in Endosomal Dynamics. Hum. Mol. Genet..

[110] Morishita S., Wada N., Fukuda M., Nakamura T. (2019). Rab5 Activation on Macropinosomes Requires ALS2, and Subsequent Rab5 Inactivation through ALS2 Detachment Requires Active Rab7. FEBS Lett..

[111] Glick D., Barth S., Macleod K.F. (2010). Autophagy: Cellular and Molecular Mechanisms. J. Pathol..

[112] Parzych K.R., Klionsky D.J. (2014). An Overview of Autophagy: Morphology, Mechanism, and Regulation. Antioxid. Redox signaling.

[113] The Interplay Between Oxidative Stress and Autophagy: Focus on the Development of Neurological Diseases-PMC. https://www.ncbi.nlm.nih.gov/pmc/articles/PMC8799983/.

[114] Filomeni G., De Zio D., Cecconi F. (2015). Oxidative Stress and Autophagy: The Clash between Damage and Metabolic Needs. Cell Death Differ..

[115] Cai H., Lin X., Xie C., Laird F.M., Lai C., Wen H., Chiang H.-C., Shim H., Farah M.H., Hoke A. (2005). Loss of ALS2 Function Is Insufficient to Trigger Motor Neuron Degeneration in Knock-Out Mice But Predisposes Neurons to Oxidative Stress. J. Neurosci..

[116] Yun H.R., Jo Y.H., Kim J., Shin Y., Kim S.S., Choi T.G. (2020). Roles of Autophagy in Oxidative Stress. Int. J. Mol. Sci..

[117] Cannariato M., Miceli M., Cavaglià M., Deriu M.A. (2021). Prediction of Protein-Protein Interactions Between Alsin DH/PH and Rac1 and Resulting Protein Dynamics. Front. Mol. Neurosci..

[118] Gautam M., Jara J.H., Sekerkova G., Yasvoina M.V., Martina M., Özdinler P.H. (2016). Absence of Alsin Function Leads to Corticospinal Motor Neuron Vulnerability via Novel Disease Mechanisms. Hum. Mol. Genet..

[119] Yoganathan S., Kumar M., Aaron R., Rangan S.R., Umakant B.S., Thomas M., Oommen S.P., Danda S. (2024). Phenotype and Genotype of Children with ALS2 Gene-Related Disorder. Neuropediatrics.

[120] Sprute R., Jergas H., Ölmez A., Alawbathani S., Karasoy H., Dafsari H.S., Becker K., Daimagüler H.-S., Nürnberg P., Muntoni F. (2021). Genotype-Phenotype Correlation in Seven Motor Neuron Disease Families with Novel ALS2 Mutations. Am. J. Med. Genet. A.

[121] van Blitterswijk M., Mullen B., Heckman M.G., Baker M.C., DeJesus-Hernandez M., Brown P.H., Murray M.E., Hsiung G.-Y.R., Stewart H., Karydas A.M. (2014). Ataxin-2 as Potential Disease Modifier in C9ORF72 Expansion Carriers. Neurobiol. Aging.

[122] Wijegunawardana D., Nayak A., Vishal S.S., Venkatesh N., Gopal P.P. (2024). Ataxin-2 Polyglutamine Expansions Aberrantly Sequester TDP-43 Ribonucleoprotein Condensates Disrupting mRNA Transport and Local Translation in Neurons. Dev. Cell.

[123] Laffita-Mesa J.M., Paucar M., Svenningsson P. (2021). Ataxin-2 Gene: A Powerful Modulator of Neurological Disorders. Curr. Opin. Neurol..

[124] Mercuri E., Sumner C.J., Muntoni F., Darras B.T., Finkel R.S. (2022). Spinal Muscular Atrophy. Nat. Rev. Dis. Primers.

[125] Spinal Muscular Atrophy|National Institute of Neurological Disorders and Stroke. https://www.ninds.nih.gov/health-information/disorders/spinal-muscular-atrophy.

[126] Rizvi S.B., Ahmed H., Zaman A., Ali A.M.N., Shah H.H., Rauf S.A., Dave T. (2024). Spinal Muscular Atrophy Type 1: A Fatal Case in a 1-Year-Old Girl with Delayed Diagnosis. Clin. Case Rep..

[127] Cances C., Vlodavets D., Comi G.P., Masson R., Mazurkiewicz-Bełdzińska M., Saito K., Zanoteli E., Dodman A., El-Khairi M., Gorni K. (2022). Natural History of Type 1 Spinal Muscular Atrophy: A Retrospective, Global, Multicenter Study. Orphanet J. Rare Dis..

[128] Cancès C., Richelme C., Barnerias C., Espil C. (2020). Clinical Features of Spinal Muscular Atrophy (SMA) Type 2. Arch. Pediatr..

[129] Salort-Campana E., Quijano-Roy S. (2020). Clinical Features of Spinal Muscular Atrophy (SMA) Type 3 (Kugelberg-Welander Disease). Arch. Pediatr..

[130] Souza P.V.S., Pinto W.B.V.R., Ricarte A., Badia B.M.L., Seneor D.D., Teixeira D.T., Caetano L., Gonçalves E.A., Chieia M.a.T., Farias I.B. (2021). Clinical and Radiological Profile of Patients with Spinal Muscular Atrophy Type 4. Eur. J. Neurol..

[131] Germain-Desprez D., Brun T., Rochette C., Semionov A., Rouget R., Simard L.R. (2001). The SMN Genes Are Subject to Transcriptional Regulation during Cellular Differentiation. Gene.

[132] Prior T.W., Krainer A.R., Hua Y., Swoboda K.J., Snyder P.C., Bridgeman S.J., Burghes A.H.M., Kissel J.T. (2009). A Positive Modifier of Spinal Muscular Atrophy in the SMN2 Gene. Am. J. Hum. Genet..

[133] SMN1 Survival of Motor Neuron 1, Telomeric [Homo Sapiens (Human)]-Gene-NCBI. https://www.ncbi.nlm.nih.gov/gene/6606.

[134] Workman E., Kolb S.J., Battle D.J. (2012). Spliceosomal Small Nuclear Ribonucleoprotein Biogenesis Defects and Motor Neuron Selectivity in Spinal Muscular Atrophy. Brain Res..

[135] Fallini C., Bassell G.J., Rossoll W. (2012). Spinal Muscular Atrophy: The Role of SMN in Axonal mRNA Regulation. Brain Res..

[136] Donlin-Asp P.G., Fallini C., Campos J., Chou C.-C., Merritt M.E., Phan H.C., Bassell G.J., Rossoll W. (2017). The Survival of Motor Neuron Protein Acts as a Molecular Chaperone for mRNP Assembly. Cell Rep..

[137] Karafoulidou E., Kesidou E., Theotokis P., Konstantinou C., Nella M.-K., Michailidou I., Touloumi O., Polyzoidou E., Salamotas I., Einstein O. (2024). Systemic LPS Administration Stimulates the Activation of Non-Neuronal Cells in an Experimental Model of Spinal Muscular Atrophy. Cells.

[138] Chaytow H., Huang Y.-T., Gillingwater T.H., Faller K.M.E. (2018). The Role of Survival Motor Neuron Protein (SMN) in Protein Homeostasis. Cell Mol. Life Sci..

[139] Jedrzejowska M., Madej-Pilarczyk A., Zimowski J., Hausmanowa-Petrusewicz I. (2006). [Pseudodominant inheritance of spinal muscular atrophy—Father and son suffering from SMA]. Neurol. Neurochir. Pol..

[140] Wirth B. (2000). An Update of the Mutation Spectrum of the Survival Motor Neuron Gene (SMN1) in Autosomal Recessive Spinal Muscular Atrophy (SMA). Hum. Mutat..

[141] Burghes A.H.M., Beattie C.E. (2009). Spinal Muscular Atrophy: Why Do Low Levels of SMN Make Motor Neurons Sick?. Nat. Rev. Neurosci..

[142] Chemello F., Pozzobon M., Tsansizi L.I., Varanita T., Quintana-Cabrera R., Bonesso D., Piccoli M., Lanfranchi G., Giacomello M., Scorrano L. (2023). Dysfunctional Mitochondria Accumulate in a Skeletal Muscle Knockout Model of Smn1, the Causal Gene of Spinal Muscular Atrophy. Cell Death Dis..

[143] Young P.J., Day P.M., Zhou J., Androphy E.J., Morris G.E., Lorson C.L. (2002). A Direct Interaction between the Survival Motor Neuron Protein and P53 and Its Relationship to Spinal Muscular Atrophy. J. Biol. Chem..

[144] Beattie C.E., Kolb S.J. (2018). Spinal Muscular Atrophy: Selective Motor Neuron Loss and Global Defect in the Assembly of Ribonucleoproteins. Brain Res..

[145] SMN Deficiency Causes Tissue-Specific Perturbations in the Repertoire of snRNAs and Widespread Defects in Splicing–PubMed. https://pubmed.ncbi.nlm.nih.gov/18485868/.

[146] Calucho M., Bernal S., Alías L., March F., Venceslá A., Rodríguez-Álvarez F.J., Aller E., Fernández R.M., Borrego S., Millán J.M. (2018). Correlation between SMA Type and SMN2 Copy Number Revisited: An Analysis of 625 Unrelated Spanish Patients and a Compilation of 2834 Reported Cases. Neuromuscul. Disord..

[147] Dosi C., Masson R. (2024). The Impact of Three SMN2 Gene Copies on Clinical Characteristics and Effect of Disease-Modifying Treatment in Patients with Spinal Muscular Atrophy: A Systematic Literature Review. Front. Neurol..

[148] Vacchiano V., Bonan L., Liguori R., Rizzo G. (2024). Primary Lateral Sclerosis: An Overview. J. Clin. Med..

[149] Turner M.R., Talbot K. (2020). Primary Lateral Sclerosis: Diagnosis and Management. Pract. Neurol..

[150] Tartaglia M.C., Rowe A., Findlater K., Orange J.B., Grace G., Strong M.J. (2007). Differentiation between Primary Lateral Sclerosis and Amyotrophic Lateral Sclerosis: Examination of Symptoms and Signs at Disease Onset and during Follow-Up. Arch. Neurol..

[151] Yang Y., Zhang L., Lynch D.R., Lukas T., Ahmeti K., Sleiman P.M.A., Ryan E., Schadt K.A., Newman J.H., Deng H.-X. (2016). Compound Heterozygote Mutations in SPG7 in a Family with Adult-Onset Primary Lateral Sclerosis. Neurol. Genet..

[152] Panzeri C., De Palma C., Martinuzzi A., Daga A., De Polo G., Bresolin N., Miller C.C., Tudor E.L., Clementi E., Bassi M.T. (2006). The First ALS2 Missense Mutation Associated with JPLS Reveals New Aspects of Alsin Biological Function. Brain.

[153] Agarwal S., Highton-Williamson E., Caga J., Matamala J.M., Dharmadasa T., Howells J., Zoing M.C., Shibuya K., Geevasinga N., Vucic S. (2018). Primary Lateral Sclerosis and the Amyotrophic Lateral Sclerosis-Frontotemporal Dementia Spectrum. J. Neurol..

[154] Miceli M., Exertier C., Cavaglià M., Gugole E., Boccardo M., Casaluci R.R., Ceccarelli N., De Maio A., Vallone B., Deriu M.A. (2022). ALS2-Related Motor Neuron Diseases: From Symptoms to Molecules. Biology.

[155] Lesca G., Eymard-Pierre E., Santorelli F.M., Cusmai R., Di Capua M., Valente E.M., Attia-Sobol J., Plauchu H., Leuzzi V., Ponzone A. (2003). Infantile Ascending Hereditary Spastic Paralysis (IAHSP): Clinical Features in 11 Families. Neurology.

[156] Cai H., Shim H., Lai C., Xie C., Lin X., Yang W.J., Chandran J. (2008). ALS2/Alsin Knockout Mice and Motor Neuron Diseases. Neurodegener. Dis..

[157] Zhang Q., Yang Q., Luo J., Zhou X., Yi S., Tan S., Qin Z. (2024). Clinical Features and Molecular Genetic Investigation of Infantile-Onset Ascending Hereditary Spastic Paralysis (IAHSP) in Two Chinese Siblings Caused by a Novel Splice Site ALS2 Variation. BMC Med. Genomics.

[158] Eymard-Pierre E., Lesca G., Dollet S., Santorelli F.M., di Capua M., Bertini E., Boespflug-Tanguy O. (2002). Infantile-Onset Ascending Hereditary Spastic Paralysis Is Associated with Mutations in the Alsin Gene. Am. J. Hum. Genet..

[159] Murala S., Nagarajan E., Bollu P.C. (2021). Hereditary Spastic Paraplegia. Neurol. Sci..

[160] Meyyazhagan A., Orlacchio A. (2022). Hereditary Spastic Paraplegia: An Update. Int. J. Mol. Sci..

[161] Varghaei P., Estiar M.A., Ashtiani S., Veyron S., Mufti K., Leveille E., Yu E., Spiegelman D., Rioux M.-F., Yoon G. (2022). Genetic, Structural and Clinical Analysis of Spastic Paraplegia 4. Parkinsonism Relat. Disord..

[162] Parodi L., Rydning S.L., Tallaksen C., Durr A. (2019). Spastic Paraplegia 4. GeneReviews^®^ [Internet].

[163] Denton K.R., Lei L., Grenier J., Rodionov V., Blackstone C., Li X.-J. (2014). Loss of Spastin Function Results in Disease-Specific Axonal Defects in Human Pluripotent Stem Cell-Based Models of Hereditary Spastic Paraplegia. Stem Cells.

[164] Wali G., Liyanage E., Blair N.F., Sutharsan R., Park J.-S., Mackay-Sim A., Sue C.M. (2020). Oxidative Stress-Induced Axon Fragmentation Is a Consequence of Reduced Axonal Transport in Hereditary Spastic Paraplegia SPAST Patient Neurons. Front. Neurosci..

[165] Solowska J.M., Baas P.W. (2015). Hereditary Spastic Paraplegia SPG4: What Is Known and Not Known about the Disease. Brain.

[166] Sauter S., Miterski B., Klimpe S., Bönsch D., Schöls L., Visbeck A., Papke T., Hopf H.C., Engel W., Deufel T. (2002). Mutation Analysis of the Spastin Gene (SPG4) in Patients in Germany with Autosomal Dominant Hereditary Spastic Paraplegia. Hum. Mutat..

[167] Casari G., Marconi R., Adam M.P., Feldman J., Mirzaa G.M., Pagon R.A., Wallace S.E., Amemiya A. (1993). Spastic Paraplegia 7. GeneReviews^®^.

[168] Wilkinson P.A., Crosby A.H., Turner C., Bradley L.J., Ginsberg L., Wood N.W., Schapira A.H., Warner T.T. (2004). A Clinical, Genetic and Biochemical Study of SPG7 Mutations in Hereditary Spastic Paraplegia. Brain.

[169] Elleuch N., Depienne C., Benomar A., Hernandez A.M.O., Ferrer X., Fontaine B., Grid D., Tallaksen C.M.E., Zemmouri R., Stevanin G. (2006). Mutation Analysis of the Paraplegin Gene (SPG7) in Patients with Hereditary Spastic Paraplegia. Neurology.

[170] Osmanovic A., Widjaja M., Förster A., Weder J., Wattjes M.P., Lange I., Sarikidi A., Auber B., Raab P., Christians A. (2020). SPG7 Mutations in Amyotrophic Lateral Sclerosis: A Genetic Link to Hereditary Spastic Paraplegia. J. Neurol..

[171] Liewluck T., Saperstein D.S. (2015). Progressive Muscular Atrophy. Neurol. Clin..

[172] Cervenakova L., Protas I.I., Hirano A., Votiakov V.I., Nedzved M.K., Kolomiets N.D., Taller I., Park K.Y., Sambuughin N., Gajdusek D.C. (2000). Progressive Muscular Atrophy Variant of Familial Amyotrophic Lateral Sclerosis (PMA/ALS). J. Neurol. Sci..

[173] van Blitterswijk M., Vlam L., van Es M.A., van der Pol W.-L., Hennekam E.A.M., Dooijes D., Schelhaas H.J., van der Kooi A.J., de Visser M., Veldink J.H. (2012). Genetic Overlap between Apparently Sporadic Motor Neuron Diseases. PLoS ONE.

[174] Cruz S.D., Cleveland D.W. (2011). Understanding the Role of TDP-43 and FUS/TLS in ALS and Beyond. Curr. Opin. Neurobiol..

[175] Fischbeck K.H. (2016). Spinal and Bulbar Muscular Atrophy Overview. J. Mol. Neurosci..

[176] Katsuno M., Tanaka F., Adachi H., Banno H., Suzuki K., Watanabe H., Sobue G. (2012). Pathogenesis and Therapy of Spinal and Bulbar Muscular Atrophy (SBMA). Prog. Neurobiol..

[177] Katsuno M., Banno H., Suzuki K., Adachi H., Tanaka F., Sobue G. (2010). Clinical Features and Molecular Mechanisms of Spinal and Bulbar Muscular Atrophy (SBMA). Adv. Exp. Med. Biol..

[178] Spada A.L. (2022). Spinal and Bulbar Muscular Atrophy. GeneReviews^®^ [Internet].

[179] Cortes C.J., La Spada A.R. (2018). X-Linked Spinal and Bulbar Muscular Atrophy: From Clinical Genetic Features and Molecular Pathology to Mechanisms Underlying Disease Toxicity. Adv. Exp. Med. Biol..

[180] Katsuno M., Adachi H., Tanaka F., Sobue G. (2004). Spinal and Bulbar Muscular Atrophy: Ligand-Dependent Pathogenesis and Therapeutic Perspectives. J. Mol. Med..

[181] Ranganathan S., Harmison G.G., Meyertholen K., Pennuto M., Burnett B.G., Fischbeck K.H. (2009). Mitochondrial Abnormalities in Spinal and Bulbar Muscular Atrophy. Hum. Mol. Genet..

[182] Feng X., Cheng X.-T., Zheng P., Li Y., Hakim J., Zhang S.Q., Anderson S.M., Linask K., Prestil R., Zou J. (2023). Ligand-Free Mitochondria-Localized Mutant AR-Induced Cytotoxicity in Spinal Bulbar Muscular Atrophy. Brain.

[183] Desai D., Stiene D., Song T., Sadayappan S. (2020). Distal Arthrogryposis and Lethal Congenital Contracture Syndrome—An Overview. Front. Physiol..

[184] Pakkasjärvi N., Ritvanen A., Herva R., Peltonen L., Kestilä M., Ignatius J. (2006). Lethal Congenital Contracture Syndrome (LCCS) and Other Lethal Arthrogryposes in Finland—An Epidemiological Study. Am. J. Med. Genet. A.

[185] Lethal Congenital Contracture Syndrome 1-NIH Genetic Testing Registry (GTR)-NCBI. https://www.ncbi.nlm.nih.gov/gtr/conditions/C1854664/.

[186] Vuopala K., Herva R. (1994). Lethal Congenital Contracture Syndrome: Further Delineation and Genetic Aspects. J. Med. Genet..

[187] Nousiainen H.O., Kestilä M., Pakkasjärvi N., Honkala H., Kuure S., Tallila J., Vuopala K., Ignatius J., Herva R., Peltonen L. (2008). Mutations in mRNA Export Mediator GLE1 Result in a Fetal Motoneuron Disease. Nat. Genet..

[188] Narkis G., Ofir R., Landau D., Manor E., Volokita M., Hershkowitz R., Elbedour K., Birk O.S. (2007). Lethal Contractural Syndrome Type 3 (LCCS3) Is Caused by a Mutation in PIP5K1C, Which Encodes PIPKI Gamma of the Phophatidylinsitol Pathway. Am. J. Hum. Genet..

[189] Noches V., Campos-Melo D., Droppelmann C.A., Strong M.J. (2024). Epigenetics in the Formation of Pathological Aggregates in Amyotrophic Lateral Sclerosis. Front. Mol. Neurosci..

[190] Martin L.J., Wong M. (2013). Aberrant Regulation of DNA Methylation in Amyotrophic Lateral Sclerosis: A New Target of Disease Mechanisms. Neurotherapeutics.

[191] Tsekrekou M., Giannakou M., Papanikolopoulou K., Skretas G. (2024). Protein Aggregation and Therapeutic Strategies in SOD1- and TDP-43- Linked ALS. Front. Mol. Biosci..

[192] Al-Mahdawi S., Virmouni S.A., Pook M.A. (2014). The Emerging Role of 5-Hydroxymethylcytosine in Neurodegenerative Diseases. Front. Neurosci..

[193] Guo W., Naujock M., Fumagalli L., Vandoorne T., Baatsen P., Boon R., Ordovás L., Patel A., Welters M., Vanwelden T. (2017). HDAC6 Inhibition Reverses Axonal Transport Defects in Motor Neurons Derived from FUS-ALS Patients. Nat. Commun..

[194] Nguyen M.D., Boudreau M., Kriz J., Couillard-Després S., Kaplan D.R., Julien J.-P. (2003). Cell Cycle Regulators in the Neuronal Death Pathway of Amyotrophic Lateral Sclerosis Caused by Mutant Superoxide Dismutase 1. J. Neurosci..

[195] Bennett S.A., Tanaz R., Cobos S.N., Torrente M.P. (2019). Epigenetics in Amyotrophic Lateral Sclerosis: A Role for Histone Post Translational Modifications in Neurodegenerative Disease. Transl. Res..

[196] Laneve P., Tollis P., Caffarelli E. (2021). RNA Deregulation in Amyotrophic Lateral Sclerosis: The Noncoding Perspective. Int. J. Mol. Sci..

[197] Yashooa R.K., Duranti E., Conconi D., Lavitrano M., Mustafa S.A., Villa C. (2025). Mitochondrial microRNAs: Key Drivers in Unraveling Neurodegenerative Diseases. Int. J. Mol. Sci..

[198] Zheleznyakova G.Y., Voisin S., Kiselev A.V., Sällman Almén M., Xavier M.J., Maretina M.A., Tishchenko L.I., Fredriksson R., Baranov V.S., Schiöth H.B. (2013). Genome-Wide Analysis Shows Association of Epigenetic Changes in Regulators of Rab and Rho GTPases with Spinal Muscular Atrophy Severity. Eur. J. Hum. Genet..

[199] Lunke S., El-Osta A. (2009). The Emerging Role of Epigenetic Modifications and Chromatin Remodeling in Spinal Muscular Atrophy. J. Neurochem..

[200] Marasco L.E., Dujardin G., Sousa-Luís R., Liu Y.H., Stigliano J., Nomakuchi T., Proudfoot N.J., Krainer A.R., Kornblihtt A.R. (2022). Counteracting Chromatin Effects of a Splicing-Correcting Antisense Oligonucleotide Improves Its Therapeutic Efficacy in Spinal Muscular Atrophy. Cell.

[201] Coppedè F. (2020). Epigenetics of Neuromuscular Disorders. Epigenomics.

[202] Riluzole: Package Insert/Prescribing Information. https://www.drugs.com/pro/riluzole.html.

[203] Wang S.-J., Wang K.-Y., Wang W.-C. (2004). Mechanisms Underlying the Riluzole Inhibition of Glutamate Release from Rat Cerebral Cortex Nerve Terminals (Synaptosomes). Neuroscience.

[204] Saitoh Y., Takahashi Y. (2020). Riluzole for the Treatment of Amyotrophic Lateral Sclerosis. Neurodegener. Dis. Manag..

[205] Roch-Torreilles I., Camu W., Hillaire-Buys D. (2000). Adverse efects of riluzole (Rilutek) in the treatment of amyotrophic lateral sclerosis. Therapie.

[206] Cantara S., Simoncelli G., Ricci C. (2024). Antisense Oligonucleotides (ASOs) in Motor Neuron Diseases: A Road to Cure in Light and Shade. Int. J. Mol. Sci..

[207] Cerillo J.L., Parmar M. (2025). Tofersen. StatPearls.

[208] Neil E.E., Bisaccia E.K. (2019). Nusinersen: A Novel Antisense Oligonucleotide for the Treatment of Spinal Muscular Atrophy. J. Pediatr. Pharmacol. Ther..

[209] Ogbonmide T., Rathore R., Rangrej S.B., Hutchinson S., Lewis M., Ojilere S., Carvalho V., Kelly I. (2023). Gene Therapy for Spinal Muscular Atrophy (SMA): A Review of Current Challenges and Safety Considerations for Onasemnogene Abeparvovec (Zolgensma). Cureus.

[210] Paik J. (2022). Risdiplam: A Review in Spinal Muscular Atrophy. CNS Drugs.

[211] Finkel R.S., Hughes S.H., Parker J., Civitello M., Lavado A., Mefford H.C., Mueller L., Kletzl H. (2025). Risdiplam for Prenatal Therapy of Spinal Muscular Atrophy. N. Engl. J. Med..

[212] Kaneko K., Hoskin J., Hodis B. (2025). Primary Lateral Sclerosis. StatPearls.

[213] McDermott C.J., Taylor R.W., Hayes C., Johnson M., Bushby K.M.D., Turnbull D.M., Shaw P.J. (2003). Investigation of Mitochondrial Function in Hereditary Spastic Paraparesis. Neuroreport.

[214] Bellofatto M., De Michele G., Iovino A., Filla A., Santorelli F.M. (2019). Management of Hereditary Spastic Paraplegia: A Systematic Review of the Literature. Front. Neurol..

[215] Lim W.F., Forouhan M., Roberts T.C., Dabney J., Ellerington R., Speciale A.A., Manzano R., Lieto M., Sangha G., Banerjee S. (2021). Gene Therapy with AR Isoform 2 Rescues Spinal and Bulbar Muscular Atrophy Phenotype by Modulating AR Transcriptional Activity. Sci. Adv..

[216] Rossi Sebastiano M., Ermondi G., Sato K., Otomo A., Hadano S., Caron G. (2022). Personalized Treatment for Infantile Ascending Hereditary Spastic Paralysis Based on In Silico Strategies. Molecules.

[217] Zou Z.-Y., Liu C.-Y., Che C.-H., Huang H.-P. (2016). Toward Precision Medicine in Amyotrophic Lateral Sclerosis. Ann. Transl. Med..

[218] Mishra J., Bhatti G.K., Sehrawat A., Singh C., Singh A., Reddy A.P., Reddy P.H., Bhatti J.S. (2022). Modulating Autophagy and Mitophagy as a Promising Therapeutic Approach in Neurodegenerative Disorders. Life Sci..

[219] Bresciani G., Manai F., Davinelli S., Tucci P., Saso L., Amadio M. (2023). Novel Potential Pharmacological Applications of Dimethyl Fumarate—an Overview and Update. Front. Pharmacol..

[220] Mayer C., Riera-Ponsati L., Kauppinen S., Klitgaard H., Erler J.T., Hansen S.N. (2024). Targeting the NRF2 Pathway for Disease Modification in Neurodegenerative Diseases: Mechanisms and Therapeutic Implications. Front. Pharmacol..

[221] Boros B.D., Schoch K.M., Kreple C.J., Miller T.M. (2022). Antisense Oligonucleotides for the Study and Treatment of ALS. Neurotherapeutics.

